# Future energy: in search of a scenario reflecting current and future pressures and trends

**DOI:** 10.1007/s10018-021-00339-1

**Published:** 2022-02-16

**Authors:** Jennifer Morris, David Hone, Martin Haigh, Andrei Sokolov, Sergey Paltsev

**Affiliations:** 1 MIT Joint Program on the Science and Policy of Global Change, Massachusetts Institute of Technology, Cambridge, MA USA; 2 Shell Scenarios Team, Shell International Ltd., London, UK

**Keywords:** Energy scenarios, Decarbonization, Energy transition, Future warming

## Abstract

Growing societal pressures, technological trends and government and industry actions are moving the world toward decarbonization and away from the “business-as-usual”. As such, the concept of a single/obvious “business as usual” or “reference” scenario is no longer relevant. Instead, there are multiple plausible futures that should be explored. We contribute one such scenario that carefully considers emission-reduction trends and actions that are likely in the future, absent globally coordinated mitigation effort. We explore the long-term implications for energy, emissions, and temperature outcomes if the world continues to address climate change in the way it has so far—through piecemeal actions and growing social and technological pressures. This *Growing Pressures* scenario results in a central scenario outcome of about 3 °C of surface temperature warming, which is higher than the “well below 2 °C” level aspired by the Paris Agreement, but lower than many widely used “no-policy” scenarios. Ongoing and growing pressures of change, the roots of which are clearly visible today, could deliver a plausible energy transition scenario to near zero emissions that plays out over the coming century. While a more aggressive transition is clearly required, this finding highlights the need to bring actions forward in time to achieve an improved outcome making use of clearly identifiable policies and technologies.

**Supplementary Information:**

The online version contains supplementary material available at 10.1007/s10018-021-00339-1.

## Introduction

The Paris Agreement has set the goal of limiting global average surface temperature warming to “well below” 2 °C (UN, 2015), and there has been a growing emphasis on limiting warming to 1.5 °C (IPCC, 2018). Much research focuses on what it would take to meet these temperature targets—the amount of emissions reductions, the required energy transition, the cost of achieving the goals, etc. Mitigation challenges are usually assessed as a comparison between a “no policy”, or “business-as-usual” scenario and the required targets (e.g. IPCC, 2014; Kriegler et al. 2014; Riahi et al. 2017; Dellink et al. 2020). The “business-as-usual” approach shapes intuition about the size of the challenge through graphical and verbal presentations contrasting the “business-as-usual” trajectory with particular temperature goals. This practice was established several decades ago, when it was relatively easy to create a “no climate policy” scenario projection because the policies and societal pressures in many regions of the world were mild or non-existent.

With growing pressure from society, more and more government and industry actions are moving the world towards decarbonization and away from the “business-as-usual”. Societal pressures and technological trends drive a reinforcing mechanism for action: pressure to pursue low-carbon solutions results in a growing array of low-carbon options, which in turn generates more pressure to employ those options. The result is changes from previously established expectations regarding “business-as-usual” development. In this context, a traditional approach of measuring mitigation efforts against some “worst case” scenario can be rather misleading. There are substantial uncertainties in how future technologies, policies and regulations, stability of nations, economic growth, and other aspects of human development will evolve, and with a curtailed resumption of global activities following the COVID-19 pandemic, these uncertainties are even greater. As such, there is no single “business as usual” scenario. Therefore, we argue that the new reality calls for moving away from traditional analysis of scenarios relative to “business-as-usual” scenarios or “references” and instead focusing on exploring multiple scenarios of plausible futures.

Indeed, the appropriateness of “no-policy” scenarios as a point of comparison for mitigation targets has been questioned, and some analysts have moved away from this practice. For example, the International Energy Agency (IEA, 2019) uses “Current Policies” and “Stated Policies” scenarios, and the United Nations Environment Programme’s Emissions Gap Report (UNEP, 2019) compares countries’ emissions-reduction pledges with global pathways that limit warming to well below 2 °C, focusing on the gap between the two. Grant et al. (2020) offer a set of scenarios for mitigation analysis, along with suggestions for the appropriate use for each. The authors argue that there are limited circumstances where a no-policy scenario is appropriate because “there is no future which does not involve substantial disruption, whether from climate policy or climate impacts”, or technological change. Therefore, a no-policy scenario represents a world which is non-existent, and comparison to such a scenario “risks overemphasizing the scale of the challenge.” Similarly, Hausfather and Peters (2020) implore people to stop using “worst case” scenarios, such as RCP8.5 from the IPCC’s Fifth Assessment Report (AR5) (IPCC, 2014), as a “business-as-usual” scenario, and to instead develop scenarios with more realistic trends.

In this paper, we add to existing sets of plausible future scenarios (such as the Shared Socioeconomic Pathways (SSP) scenarios (Riahi et al., 2017)], a scenario that carefully considers emission-reduction trends and actions that are likely in the future, absent globally coordinated mitigation effort. Our scenario considers *growing pressures from society and future technology trends* that steer the energy system away from fossil fuels and captures current and expected future momentum across different drivers to reduce emissions and fossil fuel use. This “Growing Pressures” scenario requires making assumptions about how social, political, business, technological, and other trends will evolve over time, taking into consideration possible actions and policies on local and national levels. In this scenario we do not impose global carbon pricing as is assumed in the majority prescriptive scenarios to achieve particular climate targets, such as 2 °C or 1.5 °C (MIT Joint Program, 2021; Paltsev et al., 2021a, b; IPCC, 2014). While we support the notion that global carbon pricing is widely viewed as the most efficient way of addressing global climate change, the current trends in global climate policy allude to a good chance that there will never be a truly global carbon price.

Over the last decades, the world has seen an array of fragmented policies, regulations, technology developments, business commitments and social pressures. At the same time, globally coordinated climate policy designed to achieve “well below 2 °C” (e.g., via global carbon pricing) is still largely absent. In light of this, we create a scenario that explores the following question: If the world continues to address climate change in the way it has so far (piecemeal actions and social/technological pressures that grow over time), what are the long-term implications for energy, emissions, and temperature outcomes? We present one view of a plausible estimate, along with a set of sensitivity cases.

We take a narrative approach, considering a wide variety of developments and commitments over the last decades and how we might reasonably expect those to evolve in the coming decades. The *Growing Pressures* scenario reflects the progress that has been made, and highlights the need to bring actions forward in time to achieve an improved outcome. It allows an assessment of the gap between the outcomes achieved by trends we can plausibly expect into the future and the 2 °C and 1.5 °C goals the world has set for itself.

Our *Growing Pressures* narrative results in a central scenario outcome of around 3 °C of surface temperature warming, which is not sufficient to achieve the long-term goals of the Paris Agreement, but it also does not lead to higher outcome results given the current state and pace of the energy transition and pressures from society. It should be noted that our projected path is defined by continued and growing societal pressure and action on the climate change threat, not complacency. As such, it presents a roadmap of an energy transition that could be further accelerated in pursuit of the Paris goals to limit the increase in temperature to “well below 2 °C” relative to pre-industrial levels.

In Sect. 2, we offer a narrative behind our *Growing Pressures* scenario that considers a variety of factors that have impacted the energy system over the last decades and will continue to drive its evolution into the future. In Sect. 3, we introduce the energy-economic model that is then used in Sect. 4 to quantify the storyline. Section 5 presents the resulting implications for energy, emissions and global temperature. It also explores key sensitivities around the main narrative as well as climate-related uncertainty. In Sect. 6, we conclude.

## A 100-year narrative

**Fig. 1:**
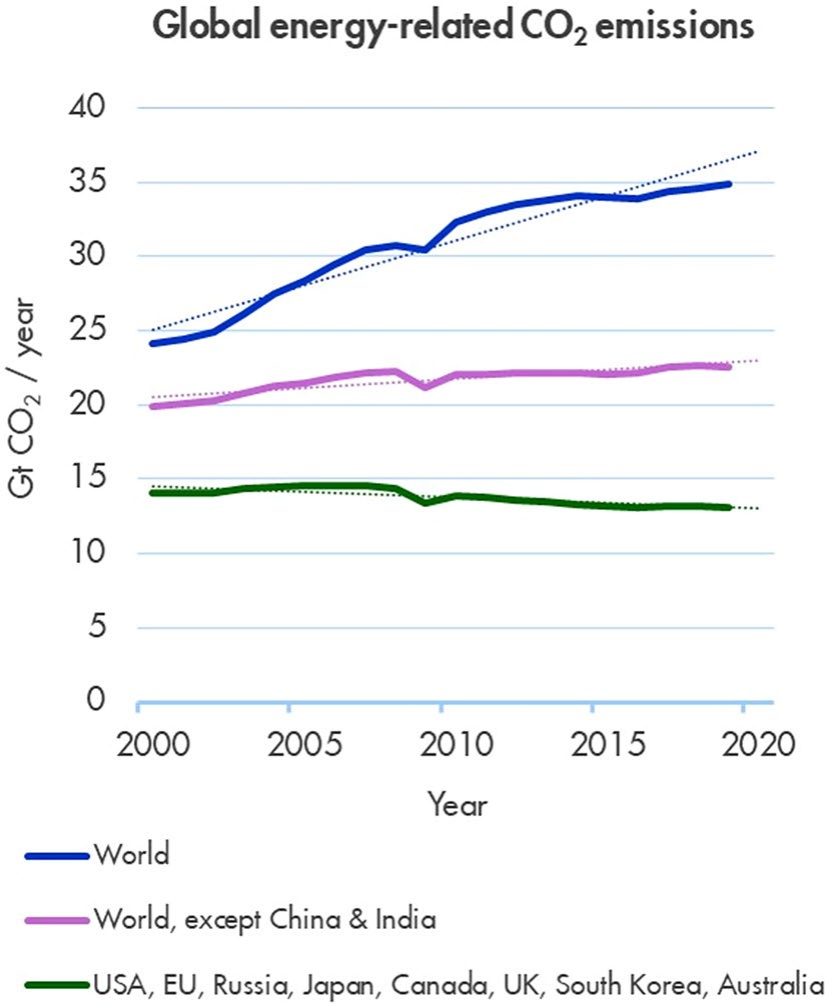
Energy-related CO_2_ emissions ( Source: Shell, 2021)

There have been changes in the energy system since the start of the twenty-first century, albeit not at anything like the pace required to meet the long-term goal of the Paris Agreement to limit average surface temperature warming to between 1.5 °C and 2 °C. Although a surge in coal use in China and India has driven the growth in global emissions, emissions growth has been less dramatic in other parts of the world, and emissions have fallen in a handful of developed countries (primarily the original Kyoto group) (see Fig. 1).

Overall, emissions growth since 2000 has been tempered by substantial growth in natural gas supply as a substitute for coal, an order of magnitude increase in non-hydro renewable electricity generation (but still representing only 10% of global generation), a significant cost reduction in solar PV and wind technologies, the arrival of the electric car and some large-scale grid battery storage. Other developments are emerging within the areas of hydrogen production and use and electrification of industrial processes, but these are not yet substantive on a global scale. More distant opportunities may exist with synthetic fuels and the known unknown remains nuclear fusion. The sum total of all these changes may be modest over the past twenty years, but the transition started from a very small base. It is now gathering pace such that over the course of the next 100 years very substantial change is expected.

Unlike any other issue that society has confronted, the physical reality of a changing climate has become a catalyst to drive long-term and persistent change in society and in our energy system. As the global average surface temperature rises and the impacts become increasingly visible, the need for energy transition will consistently return to the spotlight as other issues come and go. Transition will be forced by policy change, activism and business necessity to respond to changing demand patterns and consumer pressure. Many countries, states, cities and companies have established long-term net-zero emission goals, and while promises may not always be kept, many actions will be carried forward. These pressures and actions could drive a transition toward near zero emissions over the course of a century or so.

Society is also dealing with changes in the way it functions. Digitalization of supply chains, services and work patterns is leading to a reorganization of the global economy and paving the way towards further electrification of the final energy mix. Some of these changes were set running during the dot-com period in the late 1990s, but the majority are manifestations of little more than a decade of change. This trend has been accelerated through necessity by the COVID-19 pandemic, but irrespective of the current push, a century of digital momentum will also bring profound change in society and very likely the energy mix it requires.

Underpinning the energy transition is a confluence of multiple important factors: (a) Climate changes (Global surface temperature continues to rise, and impacts become more apparent; Sea level keeps rising with visible consequences); (b) Activism rises (Voter pressure on cities, states and countries to develop “green” policies; Shareholders pushing companies to take on net-zero emission goals and targets); (c) Local and national governments pursue interventions (Ongoing actions under the UNFCC under the banner of the Paris Agreement and the emergence of net-zero emissions (NZE) as a framing concept; Incentives and mandates drive down the cost of new energy technologies and lead to further uptake; Large established NZE policy frameworks continue to operate (e.g. EU, California) and some new NZE policy frameworks emerge (e.g. China by 2060); (d) Technology marches on (renewable energy access becomes cheaper; developments in physics, chemistry and materials sciences (e.g. PV, storage); Rapid and broadening digitalization of society); (e) Markets rule (Financial markets distance themselves from fossil fuel investments, but particularly coal, and climate-related financial disclosures bring transparency; Demands by businesses and consumers for lower carbon footprint products and some preparedness to pay for this; Development of markets to support low-carbon investment (e.g. nature-based solutions); Alternatives to coal, oil and gas becoming increasingly competitive).

While each of these will undoubtedly vary over time, their ongoing combined effect gives rise to a scenario of continuous change and transition. Here we offer a plausible energy transition scenario that plays out over the coming century, not through globally coordinated climate policy designed to achieve 2 °C or 1.5 °C (e.g., via global carbon pricing), but through persistent piecemeal action linked to the factors outlined above. The detailed storyline behind our scenario is described in Supplementary Material.

## The model

We seek to quantify this storyline with formal energy-economic modelling and identify its implications for global temperature. To do so, we employ the MIT Integrated Global System Model (IGSM) framework, which links the Economic Projection and Policy Analysis (EPPA) model, a multi-sector, multi-region, computable general equilibrium (CGE) model of the world economy to the MIT Earth System Model (MESM) of intermediate complexity (Chen et al., 2016; Paltsev et al., 2005; Sokolov et al., 2018). EPPA determines the amount of emissions of greenhouse gases (GHGs) and other pollutants associated with human activity, which is then passed to MESM to determine the implications of those emissions for temperature.

The EPPA model represents 18 regions of the world and a number of sectors, including those related to fossil fuel extraction, energy-intensive industries, other manufacturing, services, transportation, electricity generation, agriculture and households (see Supplementary Material for more information about the model). Many low- and zero-carbon options are represented in the model, including a suite of electricity generation technologies such as wind, solar, biomass, nuclear and CCS (for information about cost and penetration rate assumptions see Morris et al. 2019a, b), liquid biofuels, and electric vehicle options for household transportation.

Different versions of the EPPA model have been formulated for targeted studies, such as decarbonization of light-duty vehicles (Ghandi and Paltsev 2020), bioenergy with carbon capture and storage (Fajardy et al., 2021), use of natural gas and oil as feedstocks (Kapsalyamova and Paltsev, 2020), options for emission reduction in the hard-to-abate industrial sectors (Paltsev et al., 2021a, b), scenarios for carbon capture and storage (CCS) deployment (Morris et al., 2021a), outlook for energy, managed resources and policy prospects (MIT Joint Program, 2021) and others. For this paper, additional electricity-based technology options were added to the industrial and commercial transportation sectors, as well as for final and intermediate demand.

For its base year data, the EPPA model uses the GTAP dataset (Narayanan et al., 2012), which provides a consistent representation of energy markets as well as detailed data on regional production, consumption, and bilateral trade flows. The model is calibrated to economic and energy data from IMF (2019) and IEA (2019) for 2010 and 2015 and then it solves in 5-year time steps, traditionally to 2100. This analysis commenced before the COVID-19 pandemic and does not include energy system impacts related to it. Given the 130-year time frame, we do not see this as consequential to the outcome.

**Fig. 2:**
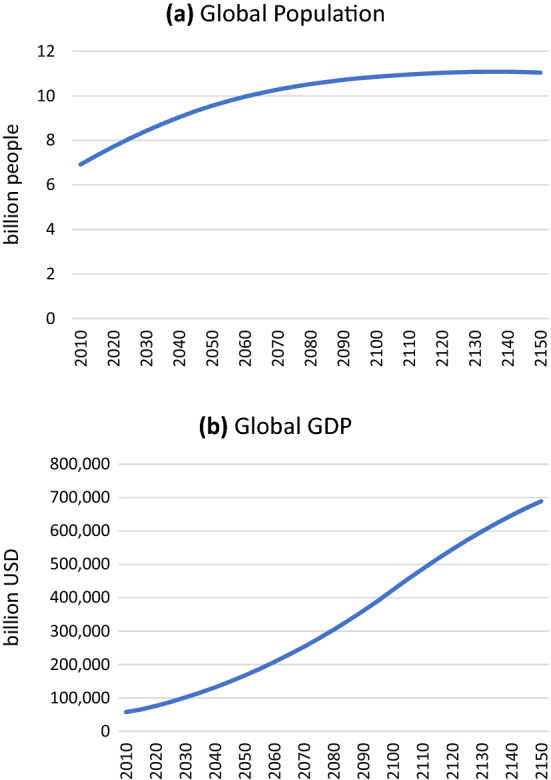
Global population (**a**) and GDP (**b**) to 2150

For this work, we extended the model horizon to 2150 to explore the potential for climate stabilization beyond 2100. To do so, we extended the default exogenous trends in EPPA for population and GDP, as well as other exogenous parameters such as autonomous energy efficiency improvements and urban pollutants. The assumed global population and GDP paths are shown in Fig. 2 (with additional detail in the Supplementary Material). For population, we use our standard assumptions to 2100 based on UN (2019) and then apply the growth rate from 2095 to 2100 to the rest of period (2100–2150). The exception is Africa, for which we slow population growth after 2100. For GDP beyond 2100, we assume it continues to grow but the growth rate slows in all regions.

As a starting point, we first ran a typical “no policy reference” scenario, which we call *Historical Trends*. This scenario does not include the Nationally Determined Contribution (NDC) targets of the Paris Agreement or any future climate policy. Scenarios like this are commonly used in the assessment of climate targets (e.g. IPCC, 2014). They result in unfettered continued use of fossil fuels even as other energy sources, such as renewables, emerge. Even scenarios that account for the Paris NDCs, but assume no further climate policy, tend to continue into the future the trends we have seen historically in terms energy and fossil fuel production and use (e.g., IEA 2019 “Stated Policies”, BP 2019, Exxon 2019, MIT 2018).

**Fig. 3:**
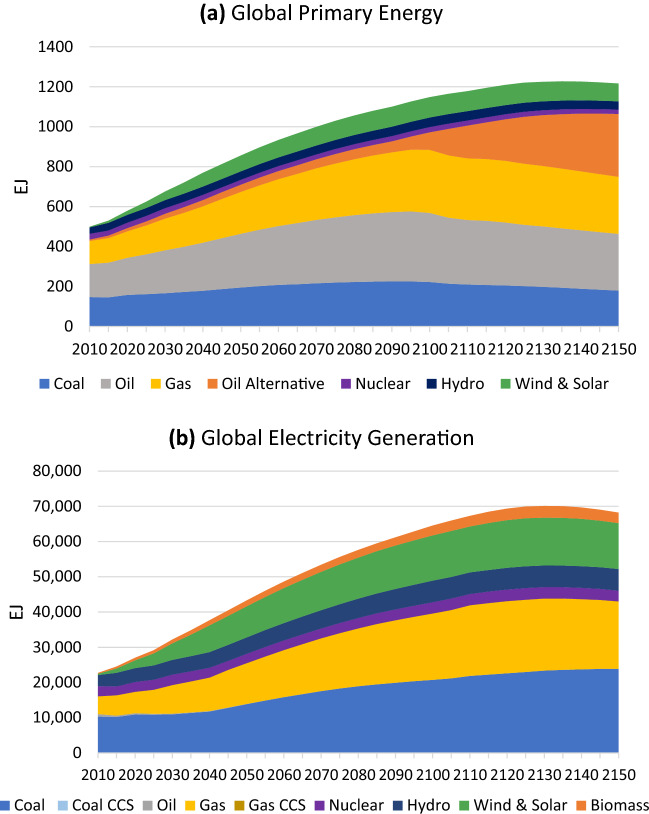
Global primary energy (**a**) and electricity generation mix (**b**) under the *Historical Trends* scenario

The primary energy and electricity generation mix associated with the *Historical Trends* scenario are shown in Fig. 3, and reflect a persistent use of fossil fuels.

**Fig. 4:**
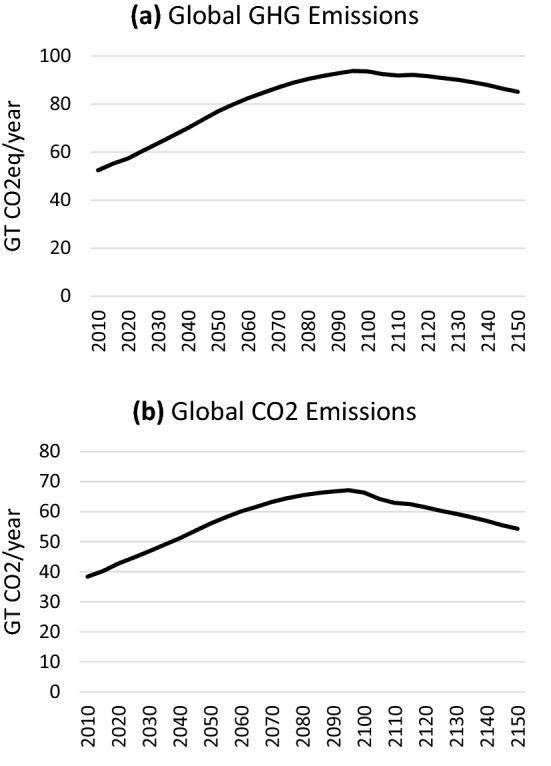
Global emissions of greenhouse gases (**a**) and CO2 (**b**) under the *Historical Trends* scenario

**Fig. 5:**
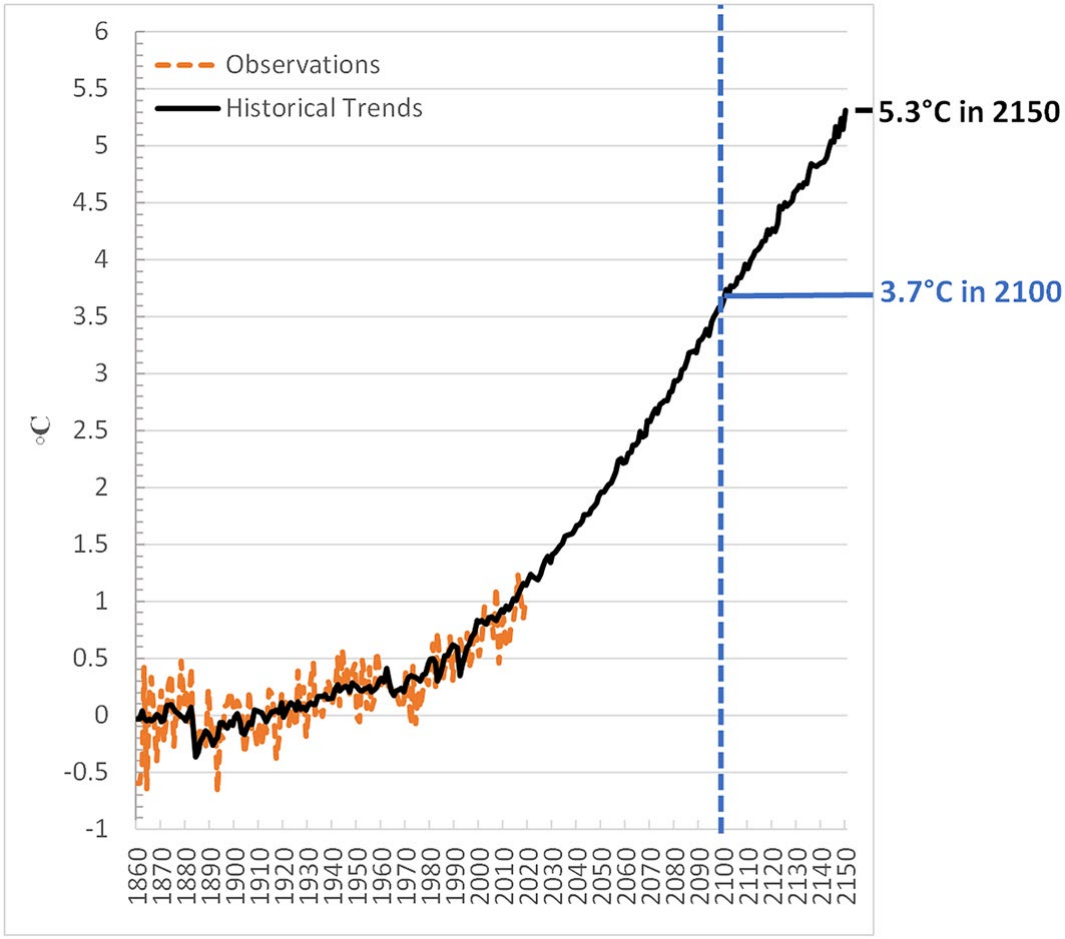
Global annual mean surface air temperature relative to pre-industrial levels (1861–1880 mean) under the *Historical Trends* scenario

The emissions associated with this scenario are shown in Fig. 4 for GHGs and for total CO2 (fossil, industrial and land use), and the resulting temperature results are shown in Fig. 5. Emissions flatten out toward the end of the century and even start to decline after 2100, reflecting primary energy use that flattens out and involves a growing share of alternatives to oil (e.g. bio-oil). This growth in oil alternatives is due to eventual oil supply constraints which increase the price of oil, allowing alternatives to compete in some regions, particularly China. Still the temperature continues to rise steadily, reaching 3.7 °C by 2100. The temperature continues to rise rapidly after 2100 (the furthest projected year for most models), reaching 5.3 °C in 2150 with still no sign of stabilizing. In the 50 years between 2100 and 2150, temperature increases an additional 1.6 °C, which is more than the total temperature increase targeted by the Paris Agreement.

This scenario presents a depressingly wide gap between where the world is headed in the absence of global policy action and the near-and long-term Paris goals. However, such a path does not account for continuing cost reductions in low- and zero-carbon technologies, persistent social pressure for climate action and green products, commitments by governments and businesses, or the increasingly difficult financing of fossil-based projects.

## Defining the *growing pressures* scenario

To quantify the narrative described in Sect. 2, we conduct a series of model runs. We start from the *Historical Trends* scenario described above, and successively add components of the narrative until we create a scenario that fully captures the narrative—the *Growing Pressures* scenario. The set of main scenario elements is described in Table 1. Consistent with the narrative above, the *Growing Pressures* scenario does not employ global carbon pricing designed to achieve the Paris goal, but rather a series of industry/country/region-level actions that lead to the shift away from fossil fuels (e.g. mandates, regulations, cost reductions, etc.). Further, we explore the transition in the absence of other significant constraints, whether on fossil resources or environmental. If either of those factors play a significant role, then they will further accelerate the transition away from fossil fuels.

**Table 1:** List of scenario elements building up to the *Growing Pressures* scenario that quantifies the narrative

Scenario element	Element description	Scenario description	Discussion
Element 1	Cost of renewables falls over time	1. Cost of renewables falls over time	Assumes that deployment driven by government policies (e.g. mandate, tax incentives, etc.) lead to these falling costs
		Cost of wind and solar falls by 1% per year, reaching a floor of 75% of their 2020 cost by 2050	
Element 2	Phase out of coal electricity	2. Element 1 + Phase out of coal electricity	The phase out of coal generation occurs on different timeframes in different regions. OECD countries are the first to stop building new coal generation. China, Africa and India are the last. Coal generation disappears globally by 2090. These phase outs are assumed to be driven by increasing difficulty securing regulatory approval and financial backing, the competitive cost of renewables, growing demands for clean air, and in some cases governmental bans
		*USA, EUR, CAN, ANZ: *no new coal electricity by 2020; no vintage coal electricity by 2040	
		*JPN:* no new coal electricity by 2030; no vintage coal electricity by 2050	
		*RUS, ROE:* no new coal electricity by 2040; no vintage coal electricity by 2080	
		*CHN:* no new coal electricity by 2045; no vintage coal electricity by 2090	
		*Rest: *no new coal electricity by 2050; no vintage coal electricity by 2090	
Element 3	Phase out of gas electricity	3. Element 1+ 2 + Phase out of gas electricity	Developed countries no longer build baseload natural gas generation capacity by 2050, and the rest of the world stopping by 2070. While some gas generation continues to be used to backup renewable generation, baseload gas generation disappears globally by 2105. These phase outs are assumed to be driven by the competitive costs renewables and energy storage, which cause the load factors for natural gas to fall to such an extent that new facilities are hardly built
		Gas generation (other than for backing up renewables) phases out	
		*Developed:* no new gas electricity by 2050; no vintage gas electricity by 2090	
		*Developing: *no new gas electricity by 2070; no vintage gas electricity by 2105	
Element 4	Energy storage solution	4. Element 1 + 2 + 3 + Energy storage solution	During the 2050s and 2060s a spate of new energy storage technologies emerges. Scale up in the 2070s in combination with solar PV and wind brings the system cost of electricity generation down further, outcompeting all other forms of generation
		Cost-competitive technology to fully resolve renewable intermittency challenges (e.g. storage) is available by 2075	
Element 5	Electrify energy-intensive industries	5. Element 1 + 2 + 3 + 4 + Electrify energy-intensive industries	Scalable solutions emerge during the 2040s in areas such as the EU for major industrial processes, such as iron ore smelting. But they take decades to deploy globally, given the slow turnover rate of big industrial facilities
		Energy-intensive industries with green technology (e.g. green electricity or hydrogen) emerge by 2050	
Element 6	Phase out of refined oil	6. Element 1 + 2 + 3 + 4 + 5 + Phase out of refined oil	Refined oil has the longest fat tail of demand. We assume electric vehicles take over for internal combustion vehicles for personal transportation by 2060 globally, aided by government policies. Urban heavy transport, such as buses, delivery vans and municipal vehicles as well as farm machinery also electrify. Long haul trucking faces greater challenges, but implements a combination of electric vehicles, biofuels and hydrogen. Aviation is the most challenging to decarbonize, and is not transformed until 2120 or beyond
		EVs take over ICEVs for personal transportation by 2060 globally	
		Carbon-free alternatives to refined oil (e.g. bio-oil, EVs, hydrogen) take over in Developed countries by 2100 and in Developing countries by 2120	
Element 7	Phase out fossil final demand	7. Element 1 + 2 + 3 + 4 + 5 + 6 + Phase out fossil in final demand	Combustion of fuels in homes begins to disappear in many urban settings from the 2040s onwards. Convenience and cost allow electric-based heating and cooking to progressively remove natural gas and heating oil from domestic use
		Globally, coal out of final demand by 2030, gas by 2060 and oil by 2090	
Element 8	Phase out fossil inputs	8. Element 1 + 2 + 3 + 4 + 5 + 6 + 7 + Phase out fossil inputs into production	All production processes and sectors eventually seek to replace fossil inputs with alternatives, mainly electricity
		Fossil energy out by 2130 globally	
Element 9	Non-CO_2_ GHG reductions	9. Element 1 + 2 + 3 + 4 + 5 + 6 + 7 + Non-CO_2_ GHG reductions	The shift away from fossil fuels throughout the economy, combined with changes in agricultural and various industrial practices as well as diets, also leads to reductions in non-CO_2_ GHGS such as CH_4_ and N_2_O
		Reductions in CH_4_ and N_2_O similar to the Shell Sky scenario	
		Growing pressures scenario	All scenario elements together create a plausible baseline energy transition scenario that captures current and expected future momentum across different drivers to reduce emissions and fossil fuel usage

*USA* United States, *EUR* European Union, *CAN* Canada, *ANZ* Australia and New Zealand, *JPN* Japan, *RUS* Russia, *ROE* Rest of Europe, *CHN* China

All EPPA regions are defined in Figure S1 in Supplementary Material. Shell Sky scenario is described in Shell (2018), see Supplementary Material for profiles of CH_4_ and N_2_O.

*Scenario Elements 1* and *4* reflect the narrative about the falling costs for renewable energy and energy storage. In *Scenario Element 1,* the cost of wind and solar technologies are assumed to fall by 1% per year, reaching a floor in 2050 of 25% below their 2020 costs. *Scenario Element 4* assumes a cost-competitive scaled energy storage technology is available by 2075, which fully resolves renewable intermittency challenges. The assumption is that deployment driven by commercial projects and fragmented policies (e.g. tax credits, renewable portfolio standards, feed-in tariffs, research and development, etc.) lead to these falling costs.

*Scenario Element 2* builds on *Scenario Element 1* and phases out^1^ coal generation, with the phase out occurring on different timeframes in different regions. OECD countries are the first to stop building new coal generation. China, Africa and India are the last. Coal generation disappears globally by 2090. These phase outs are assumed to be driven by increasing difficulty securing regulatory approval and financial backing, the competitive cost of renewables, growing demands for clean air, and in some cases governmental bans.

*Scenario Element 3* adds to *Scenario Element 2* the phase out of natural gas generation, with developed countries no longer building baseload natural gas generation capacity by 2050, and the rest of the world stopping by 2070. While some gas generation continues to be used to backup renewable generation, baseload gas generation disappears globally by 2105. These phase outs are assumed to be driven by the competitive costs renewables and energy storage, which cause the load factors for natural gas to such an extent that new facilities are hardly built.

The narrative about widespread electrification is reflected in *Scenario Elements 5, 6, 7 and 8. Scenario Element 5* focuses on energy intensive industries, assuming they begin to deploy green technology—likely electrification or hydrogen, but also potentially CCS—by 2050. The assumption is that industrial companies in the EU facing national and regional directives to reach net-zero emissions by 2050 lead the way in efforts to electrify various processes or convert them to hydrogen-based systems (e.g. electrolysis using renewable energy). These innovations spread globally as the cost of renewable energy falls. Energy-intensive industries are largely electrified by 2075 in the OECD, and by 2110 in the rest of the world.

*Scenario Element 7* incorporates the electrification of final demand, assuming that globally coal is phased out of final demand by 2030, gas by 2060 and oil by 2090 as home heating and cooking shift to electricity. *Scenario Element 8* assumes fossil fuels are phased out of intermediate demand (e.g. use as inputs into production) globally by 2130, largely being replaced by electricity.

*Scenario Element 6* focuses on the phase out of refined oil, much of which is related to transportation. We assume electric vehicles take over for internal combustion vehicles for personal transportation by 2060 globally. Urban heavy transport, such as buses, delivery vans and municipal vehicles as well as farm machinery also electrify. Long haul trucking faces greater challenges, but implements a combination of electric vehicles, biofuels and hydrogen. Aviation is the most challenging to decarbonize, and is not transformed until 2120 or beyond.

*Scenario Element 9* adds in reductions in non-CO2 GHG emissions. We assume reductions in CH4 and N2O similar to the Shell Sky scenario (Shell 2018; see Supplementary Material). These reductions are driven by the shift away from fossil fuels throughout the economy, and changes in agricultural and various industrial practices as well as diets.

## Results

### Main results

All scenario elements together create the *Growing Pressures* scenario, a plausible energy transition scenario that captures current and expected future momentum across different drivers to reduce emissions and fossil fuel usage.

**Fig. 6:**
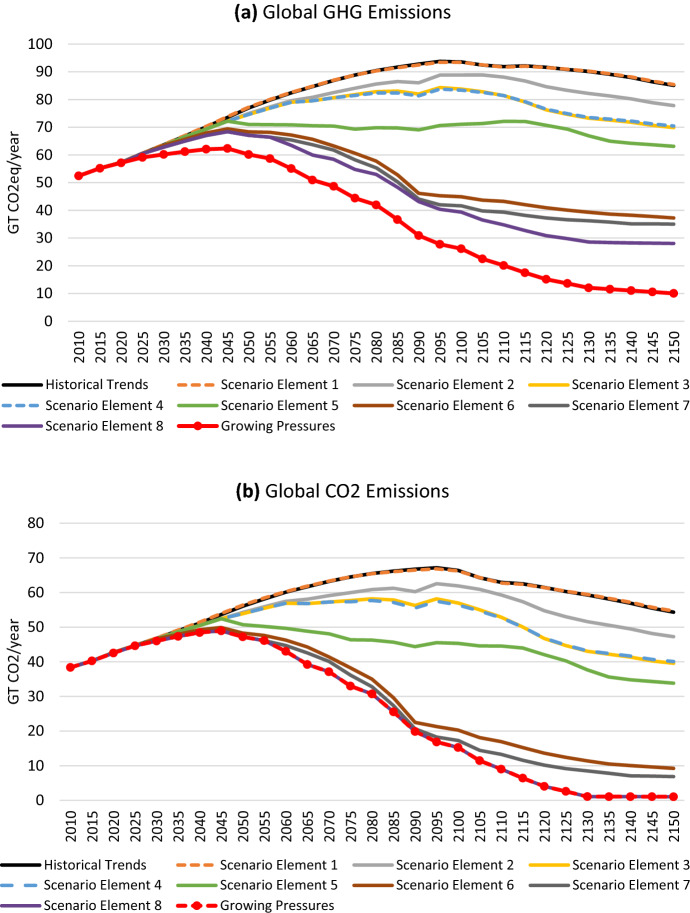
Global emissions of GHGs (**a**) and CO_2_ (**b**) in a series of scenarios building up to the *Growing Pressures* scenario described in the narrative

The GHG and CO_2_ emissions paths for each of the scenario elements are shown in Fig. 6**.** Sequentially adding additional elements of the narrative allows us to identify areas that have the largest impact on emissions reductions. The falling cost of renewables (*Scenario Element 1*) has virtually no impact on emissions. This is due to a rebound effect: cheap renewable electricity leads to less fossil fuel use in electricity, which leads to falling costs of fossil fuels and therefore more fossil fuel use in other sectors. We see the same story under *Scenario Element 4* in which cost-competitive energy storage leads to a massive increase in renewable electricity, pushing fossil fuels out of electricity generation, but increasing their use in other sectors. Figure 6 also shows that cleaning up the electricity sector alone has only a small impact on overall emissions reductions, highlighting the importance of decarbonization in other sectors. We also see that electrifying (or pursuing other low-carbon options in) energy-intensive industries (as we have imagined in *Scenario Element 5*) can lead to more emissions reductions earlier in the century. The gradual reduction in refined oil demand (*Scenario Element 6*) has by far the largest impact on emissions reductions, reflecting oil’s pervasive use throughout the global economy. Phasing fossil fuels out of final demand (*Scenario Element 7*) has a small emissions impact. Replacing fossil fuel inputs into production (*Scenario Element 8*) has a sizeable impact. These actions lead to essentially zero CO_2_ emissions by 2130. However, global GHGs are almost 30 Gt in 2130 and beyond. Adding in the assumption that non-CO_2_ GHGs (e.g. CH_4_ and N_2_O from agriculture) will also be reduced (*Scenario Element 9,* the *Growing Pressures* scenario) brings global GHG emissions to about 10 Gt—still not zero, but close.

**Fig. 7:**
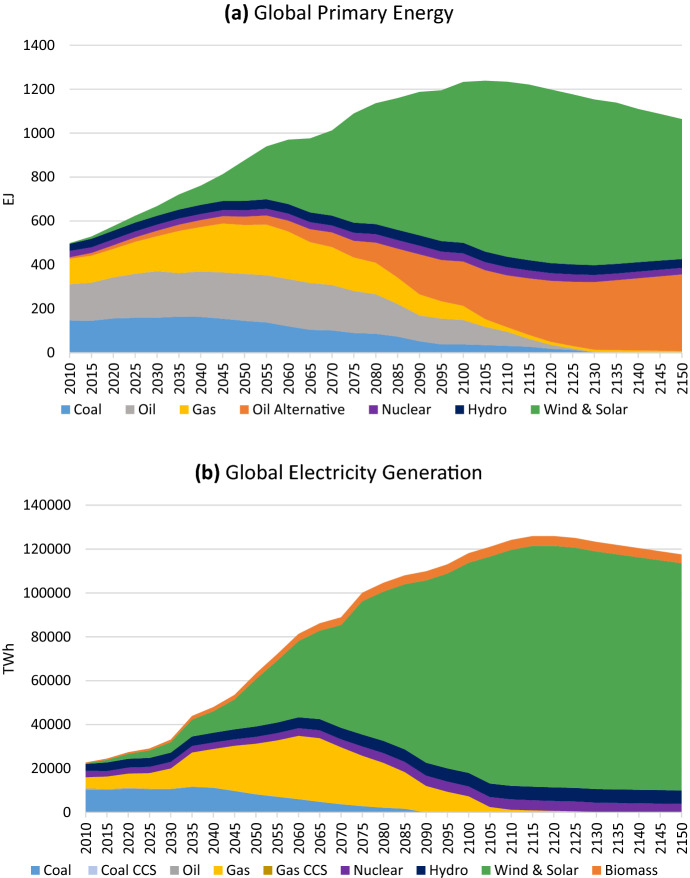
Global primary energy (**a**) and electricity generation (**b**) mix under the *Growing Pressures* scenario

The primary energy and electricity generation mixes for the final *Growing Pressures* scenario are shown in Fig. 7. Although fossil fuels ultimately disappear from the global primary energy mix by 2130, it is a long, drawn-out process, with global reductions largely taking place after 2060. “Oil Alternative”, a category representing a combination of biofuels, electrification and hydrogen which substitute for refined oil, grows after that point. There is also a massive increase in renewable energy as electrification becomes widespread, driven by global electricity systems dominated by renewable generation. The global electricity mix shows a slightly faster transition than in primary energy, and is nearly decarbonized by 2100. However, natural gas generation continues to play an important role, continuing to grow through about 2065, after which no new capacity is built, retirements of old capacity occur, and renewables with an energy storage solution become increasingly cost competitive.

**Fig. 8:**
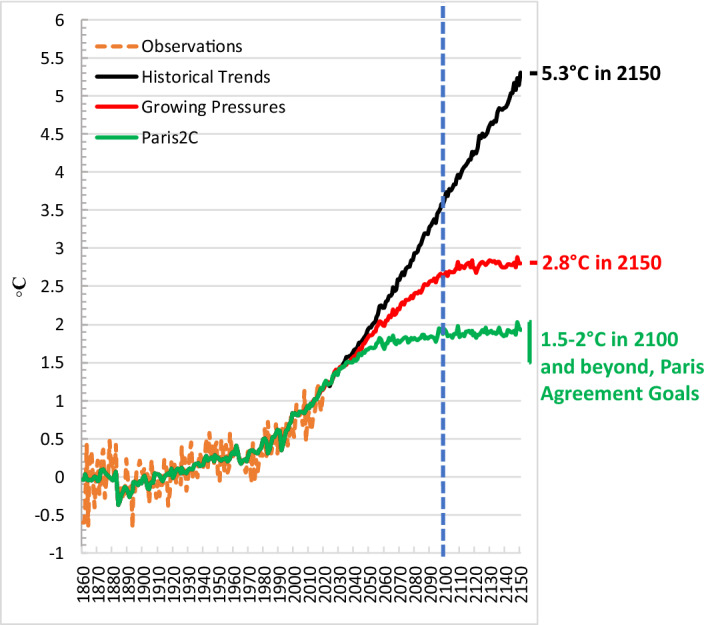
Global average surface air temperature relative to pre-industrial levels (1861–1880 mean) under the *Growing Pressures* scenario, the *Historical Trends* scenario and *Paris2C* scenario

In Fig. 8, we show the temperature implications of the *Growing Pressures* scenario compared to the *Historical Trends* scenario as well as a *Paris2C* scenario designed to meet the Paris NDC targets in 2030 and then pursue a global carbon price consistent with achieving 2 °C by 2100 with a 66% probability. Under the *Growing Pressures* scenario, the increase in global temperature stabilizes at 2.8 °C by 2150, a full 2.5 °C lower than where the *Historical Trends* scenario ends up in 2150 as it continues an upward trajectory.

**Fig. 9:**
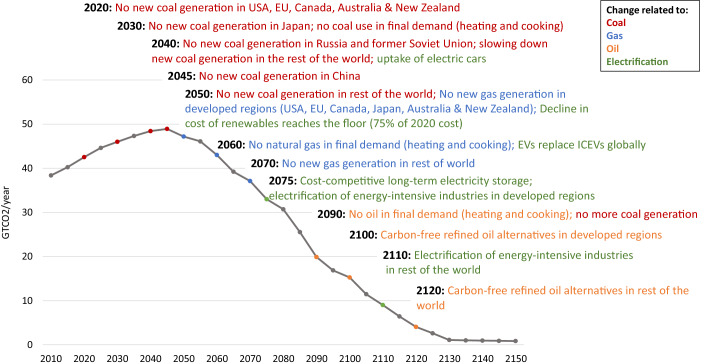
Summary of assumed actions driving the *Growing Pressures* scenario

To be clear, a lot of action needs to take place for the *Growing Pressures* scenario to be realized—a summary is provided in Fig. 9. Continued and growing social, political, business and technology pressures can drive these actions toward a low-carbon world.

**Fig. 10:**
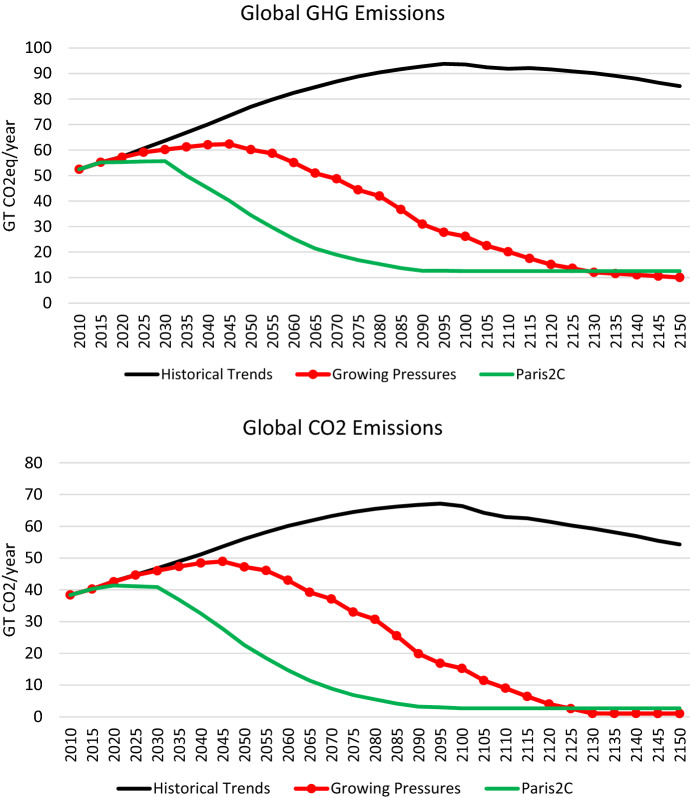
Global emissions of GHGs (**a**) and CO_2_ (**b**) under the *Growing Pressures* scenario, the *Historical Trends* scenario and *Paris2C* scenario

Of course, temperature stabilization at 2.8 °C above preindustrial levels does not match the goals of the Paris Agreement (see Figs. 8 and 10 for a comparison of temperature impacts and emissions pathways between the *Growing Pressures* scenario and the *Paris2C* scenario). However, the pathway described shows the significant progress that has been made in recent years in redefining our collective future. With the exception of aviation, the scenario makes use of a set of technologies and changes in energy use that are visible now, rather than just imagined as necessary. In addition, the path laid out in the *Growing Pressures* scenario provides a roadmap of an energy transition that could be accelerated, particularly with global coordination and carbon pricing, in order to get closer to the Paris Goals.

### Sensitivities analysis

While we formulated in Sect. 4 the main driving forces that lead to the *Growing Pressures* scenario (shown in Fig. 9), the exact timing and magnitude of those actions are subject to uncertainty. To explore the sensitivity with respect to our main assumptions, we conducted five sensitivity cases (see Table 2) related to the phase out of refined oil, the electrification of energy-intensive industries, and actions in developing regions (India and Africa).

**Table 2:** Sensitivity Scenarios: variations of the *Growing Pressures* (GP) scenario

Scenario	Description
GP_SlowOil	Slower phase out of refined oil
GP + FastOil	Faster phase out of refined oil
GP_LessEINT	Only 50% energy intensive industry electrified
GP_NoAFR	Africa takes limited action to reduce fossil fuels
GP_NoAFR_ NoIND	Africa and India take limited action to reduce out fossil fuels

As described in Sect. 4, in the main version of the *Growing Pressures* scenario we envision that developed regions will move to carbon-free alternatives to refined oil by 2100 and the rest of the world completely switches to carbon-free alternatives to refined oil by 2120. Since the phase out of refined oil is critical to the timing of the transition (see Fig. 6 for the change in emissions trajectory between *Scenario Element 5* and *Scenario Element 6*), we test alternative cases regarding the oil phase out.

In the *GP_SlowOil* scenario, we delay the phase out of refined oil and assume that even by 2150 there are pockets of the global economy that continue to rely on refined oil. In this case, development of alternatives for oil in commercial transportation (particularly in long-haul trucking, shipping, and air travel) prove to be more difficult and/or expensive than imagined in the main version of the *Growing Pressures* scenario. In contrast, in the *GP* + *FastOil* scenario the progress with oil alternatives is more advanced, and by mid-century a substantial deployment of carbon-free alternatives to refined oil is taking place. This scenario is supported by the recent announcement by major energy companies (BP, Shell, Total, and others) to search for the solutions to become “net-zero” emissions by 2050. Here we assume that other companies join the pledges and alternative options are deployed more quickly. However, given the prevalence of oil in the global economy, we assume that a faster oil phase out requires additional action beyond what might be assumed in our *Growing Pressures* scenario.

We also test a scenario where it is more difficult to remove fossil-based inputs to energy-intensive industries than imagined in the *Growing Pressures* scenario (through electrification or use of “green” hydrogen, or, alternatively, by using CCS technology to capture emissions). In this scenario (called *GP_LessEINT*), only 50% of energy intensive industry activities are electrified by 2110.

Additional scenarios explore cases in which the low-carbon transition is delayed in some developing regions. We use examples of Africa and India as populous regions with substantial low-income populations that may pursue other development objectives and stay with fossil fuels, perhaps because of domestic availability. In these cases, these regions still experience the cost declines in renewable generation and energy storage technologies, and face increasing barriers (e.g. financing costs) to fossil electricity generation. However, they do not pursue direct restrictions on fossil energy use, such as limiting the ability to purchase an internal combustion vehicle. While we consider unlikely the situation of a fragmented world where these regions would forever continue using oil and oil-based technologies while the rest of the world has moved on to newer and cleaner alternatives, these sensitivity cases provide us with useful benchmarking. As an example, to continue making use of gasoline through to the end of the century and beyond, certain countries would need to develop a domestic vehicle production industry as we envisage a world in which the current global manufacturers have opted to move on to electric vehicle technology.

**Fig. 11:**
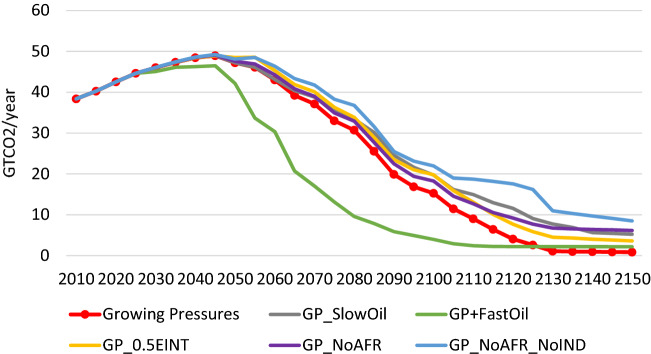
Global CO_2_ emissions in the *Growing Pressures* scenario (red line with round markers), and its alternative variations

**Fig. 12:**
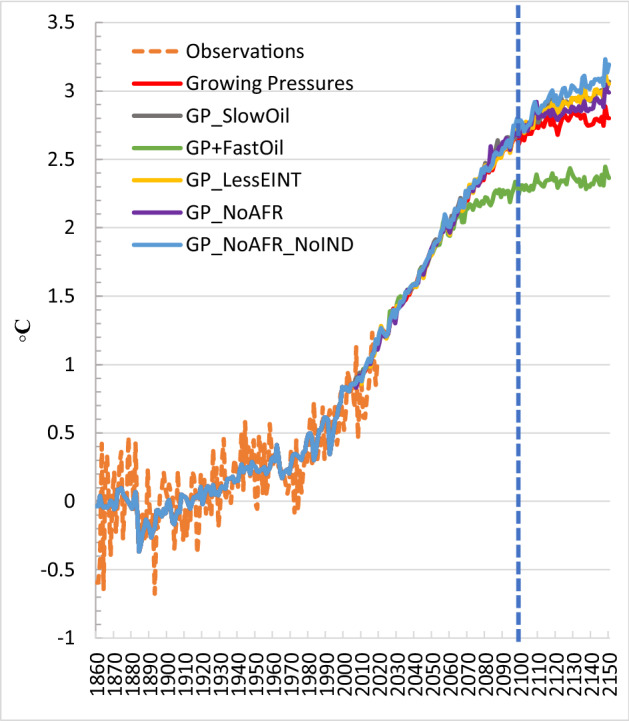
Global average surface air temperature relative to pre-industrial levels (1861–1880 mean) under the *Growing Pressures* scenario, and its alternative variations

The CO_2_ emissions paths associated with each sensitivity scenario are shown in Fig. 11, and their temperature implications are provided in Fig. 12. As expected, delays in the low-carbon transition increase the global CO_2_ profiles and the resulting temperature.

The *GP_SlowOil* scenario increases global CO_2_ emissions relative to the *Growing Pressures* scenario throughout the time horizon and as a result the temperature increase in 2150 ends up at just over 3 °C, and by that time the temperature is not stabilized but continues to rise. This is compared to a stabilization at 2.8 °C in the *Growing Pressures* scenario. In contrast, in the *GP* + *FastOil* scenario global CO_2_ emissions are significantly reduced. The emissions trajectory is slightly lower than the *Growing Pressures* scenario between 2025 and 2045 and then emissions fall dramatically through 2110, where they remain close to zero (at about 2 Gt CO_2_) through 2150. This dramatic change in emissions is reflected in the temperature increase, which stabilizes at about 2.4 °C.

In the *GP_LessEINT* scenario, emissions are increased relative to the *Growing Pressures* scenario, and as a result the global temperature increase ends up at just over 3 °C in 2150. Limited decarbonisation activities in Africa also lead to a similar temperature increase. Among the sensitivity cases that we have tested, the largest temperature increase (3.2 °C by 2150) is in the scenario when both Africa and India pursue limited decarbonisation activities.

**Fig. 13:**
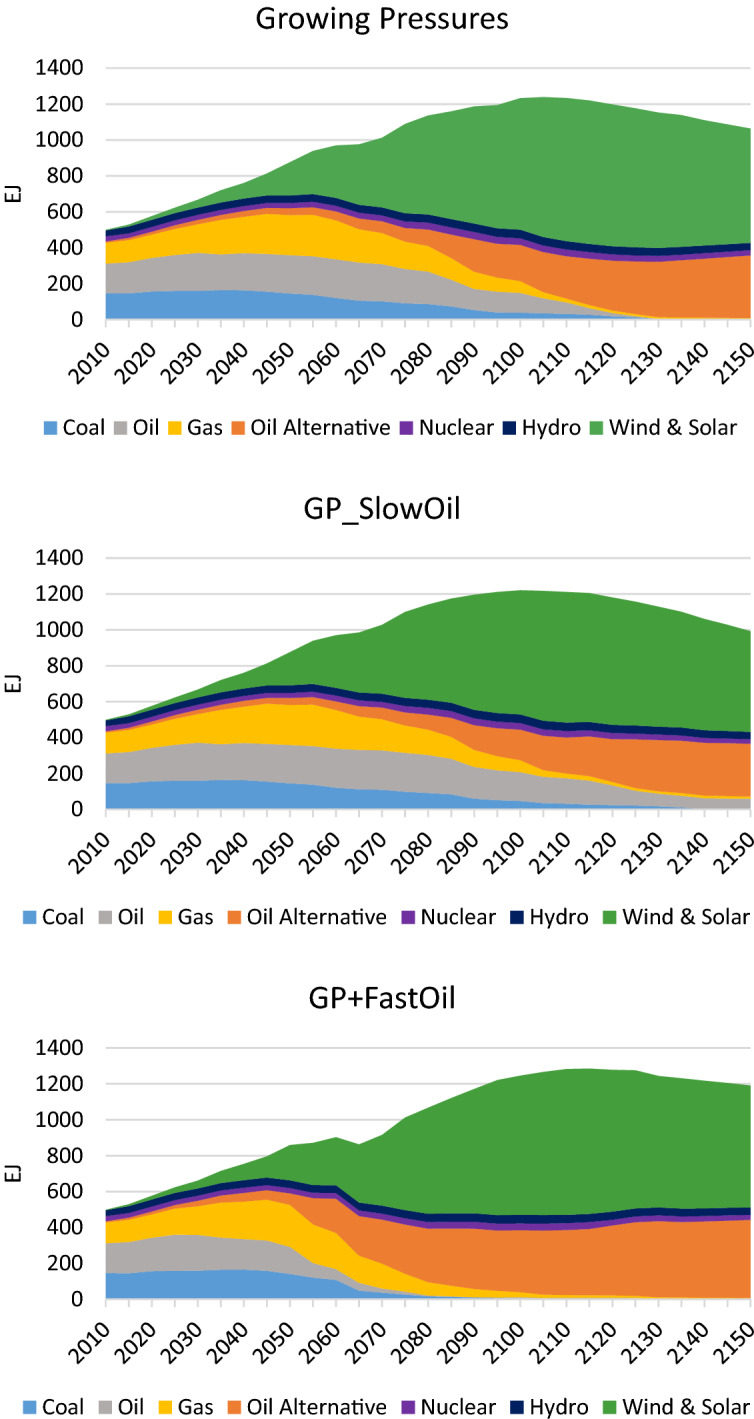
Global primary energy mix under different assumptions about the phase out of oil

The results described above are driven by the changes in the global primary energy mix. Figure 13 illustrates these changes in the cases with different phase out trajectories for refined oil. In the *Growing Pressures* scenario, oil is removed from the global energy mix in 2120, while in the *GP_SlowOil* scenario oil lingers through 2150, reflecting its continued use in some sectors (e.g. air transport) and some regions (e.g. Africa and the Middle East). In the *GP* + *FastOil* scenario, oil is significantly reduced by 2055 and phased out by 2080. These sensitivity cases indicate how important phasing out oil is to long-term climate stabilization goals. This suggests the need for more aggressive actions to develop and deploy technologies, fuels and infrastructure that enable the transition away from refined oil at a faster pace.

**Fig. 14:**
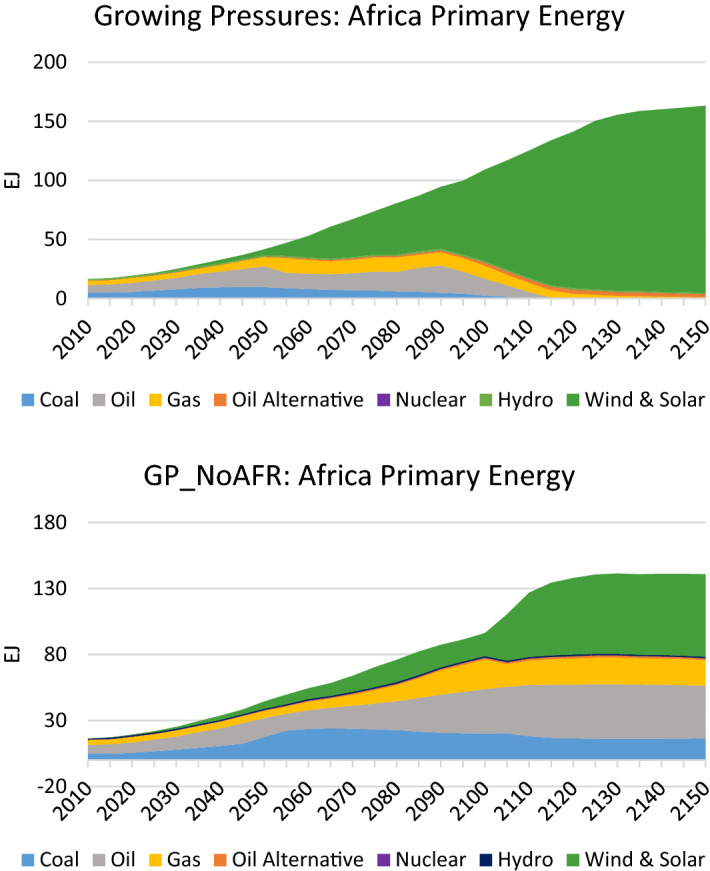
Primary energy mix in Africa in the *Growing Pressures* scenario and the *GP_NoAFR* scenario in which Africa takes limited action to reduce fossil fuels

**Fig. 15:**
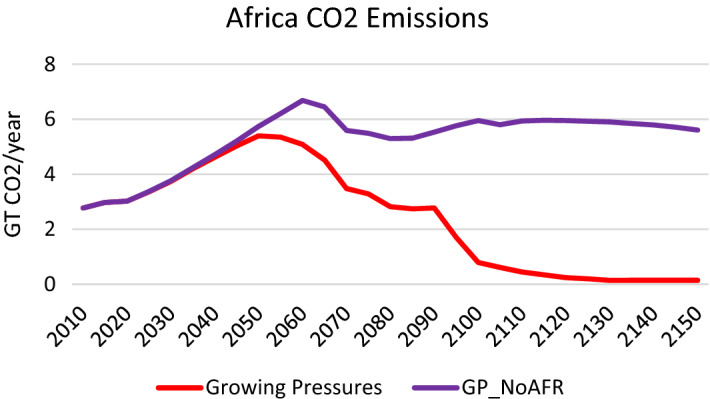
CO_2_ Emissions in Africa in the *Growing Pressures* scenario and the *GP_NoAFR* scenario in which Africa takes limited action to reduce fossil fuels

When Africa does not take aggressive decarbonisation actions, it has a starkly different primary energy mix (see Fig. 14). In comparison to the *Growing Pressures* scenario, where the continent is almost fully electrified by 2120–2130, in the *GP_No AFR* scenario it continues to employ coal, oil and gas and uses far less renewables. As a result, Africa’s emissions are significantly higher and about double from 2020 to 2150 (see Fig. 15). While in the *Growing Pressures* scenario Africa’s CO_2_ emissions approach zero after 2100, in the *GP_No AFR* scenario the emissions in 2050–2150 are relatively stable at about 6 GtCO_2_/year. With a rapidly growing population in Africa, stabilizing emissions is an important milestone. However, decarbonisation actions in Africa need to be enhanced to reach the temperature goals of the Paris Agreement.

**Fig. 16:**
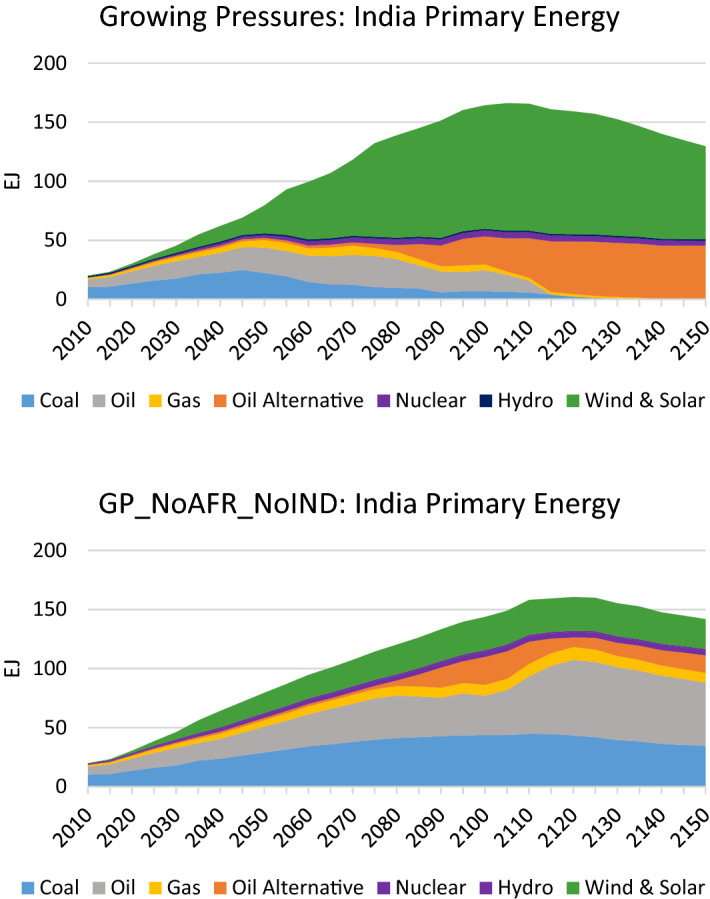
Primary energy mix in India in the *Growing Pressures* scenario and the *GP_NoAFR_NoIndia* scenario in which India and Africa take limited action to reduce fossil fuels

**Fig. 17:**
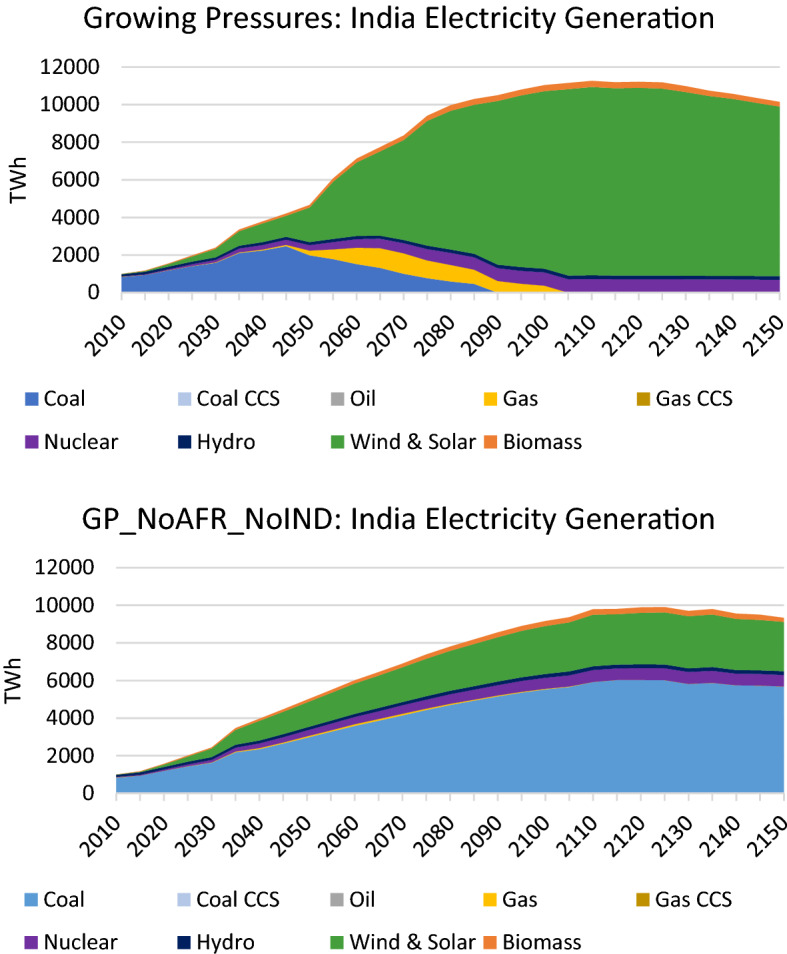
Electricity generation mix in India in the *Growing Pressures* scenario and the *GP_NoAFR_NoIndia* scenario in which India and Africa take limited action to reduce fossil fuels

**Fig. 18:**
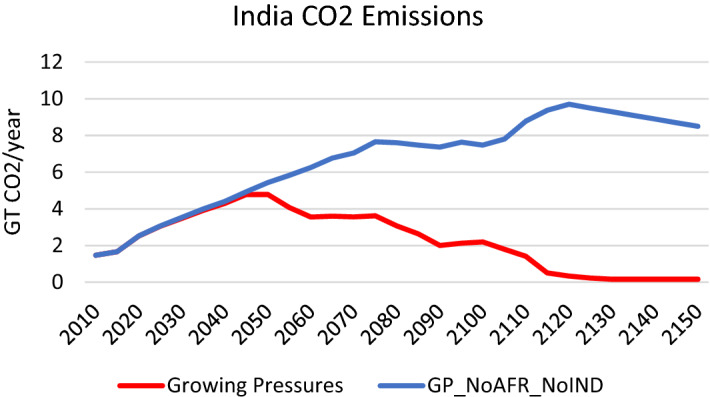
CO_2_ Emissions in India under the *Growing Pressures* scenario and the *GP_NoAFR_NoIND* scenario in which India and Africa takes limited action to reduce fossil fuels

In the case where India is slow to adopt the low-carbon technologies that are pervasive elsewhere, it continues its use of fossil fuels to an even greater extent than Africa. This is seen in its primary energy mix which uses large amounts of oil and coal (see Fig. 16), and is particularly stark in its electricity generation mix which is dominated by coal instead of renewables (see Fig. 17). These changes result in rising emissions that end up over four times higher than 2020 levels by 2150 (see Fig. 18). These differences in India, together with the differences in Africa, translate to an increase in global temperature by 3.2 °C by 2150 (as depicted in Fig. 12).

Our sensitivity analysis provides a quantification of the impacts from relaxing or tightening key assumptions in the *Growing Pressures* scenario. It shows that even with less courageous assumptions about future decarbonisation actions, the global temperature increase could be bounded to about 3 °C, not 5 °C or more (as suggested by RCP8.5-like trajectories). Our assessment also indicates areas to focus actions in order to put the world on a trajectory that could align with the long-term goals of the Paris Agreement—namely the use of refined oil, energy-intensive industries, and action in key developing regions. Faster decarbonization is those areas could put more aggressive climate stabilization targets within reach.

### Climate-related uncertainty

Temperature outcomes are not solely determined by the emissions trajectory, but also by the climate response to those emissions, which is uncertain. To account for this climate-related uncertainty, we run 400-member ensembles of the MIT Earth System Model (MESM) (Sokolov et al., 2018) based on probability distributions for climate sensitivity, ocean heat uptake and aerosol forcing (Libardoni et al, 2018). Accounting for uncertainty in the climate response to the emissions from the *Growing Pressures* scenario, gives a 90% probability bound of 2150 temperature increase of 2.24–3.51 °C (see Table 3).

**Table 3:** Global average surface air temperature relative to pre-industrial levels (1861–1880 mean) under the *Growing Pressures* scenario at the end of the century (2091–2100) and for 2141–20,150 for given percentiles when accounting for uncertainty in climate response

	Temperature at given percentiles						
	5%	17%	33%	50%	66%	83%	95%
2091–2100	2.10	2.34	2.51	2.64	2.79	2.95	3.21
2141–2150	2.24	2.48	2.66	2.80	2.98	3.22	3.51

**Fig. 19.**
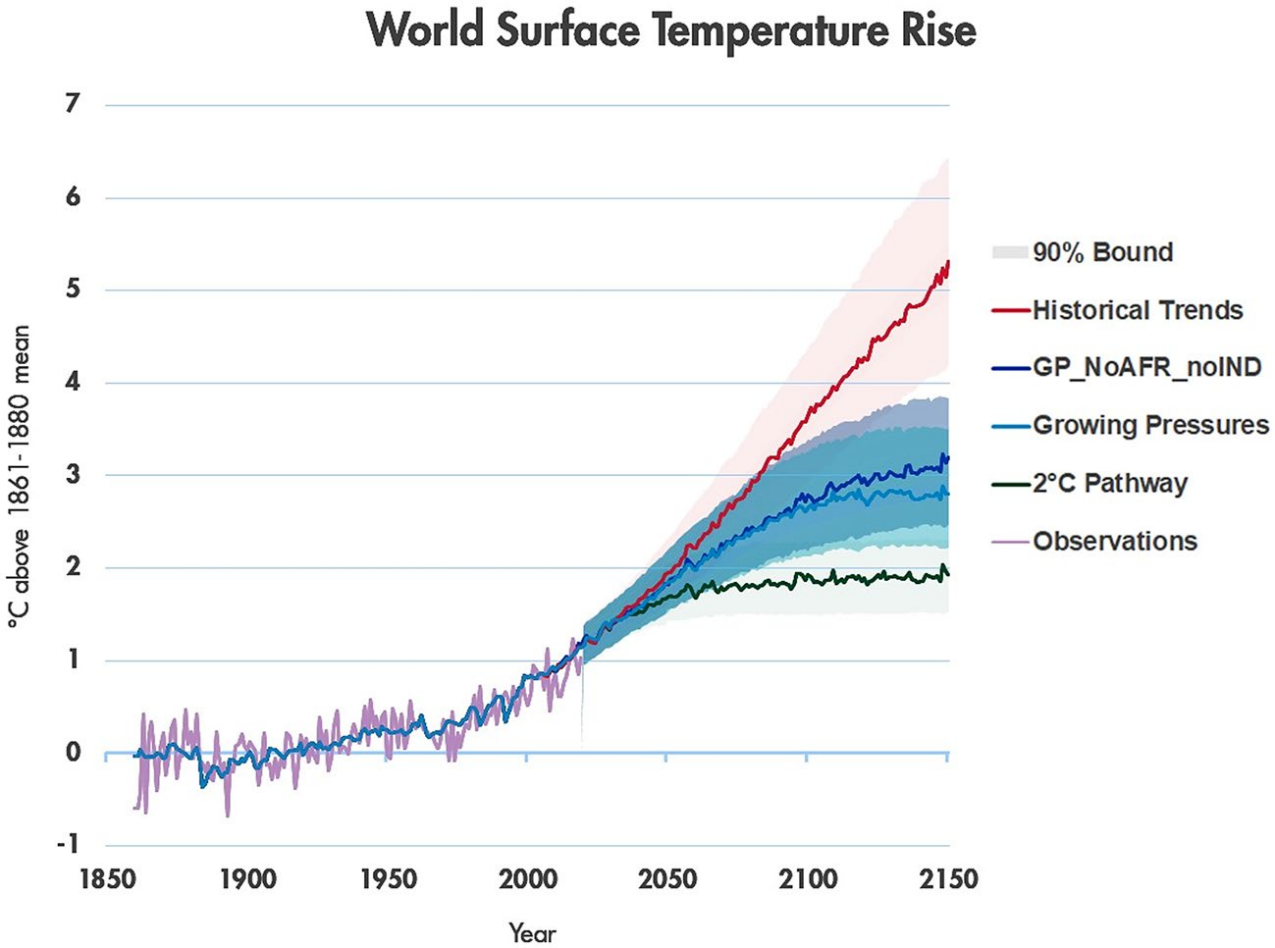
90% probability bound for temperature under the *Growing Pressures* scenario, as well as under the *GP_NoAFR_NoIND, Historical Trends* and 2C scenarios (solid lines are the median temperature outcomes)

Figure 19 shows the 90% probability bound for temperature under the *Growing Pressures* scenario, as well as under the *GP_NoAFR_NoIND, Historical Trends* and 2C scenarios. Of the alternative variations of the *Growing Pressures* scenario explored in Sect. 5.2, the *GP_NoAFR_NoIND* scenario has the highest temperature outcome (3.2 °C) given median climate parameters, and the original *Growing Pressures* scenario the lowest (2.8 °C).^2^ Figure 19 therefore provides an image of the temperature range from the *Growing Pressures* scenario accounting for both scenario uncertainty and climate uncertainty. The 2150 temperature spans a range of 2.24 °C (the lower bound of *Growing Pressures*) to 3.84 °C (the upper bound of *GP_NoAFR_NoIND*). This range is an important reminder that a given emissions trajectory cannot guarantee a temperature outcome. Rather, uncertainty in the climate system requires that actions be continually adjusted if a temperature target is to be met.

## Conclusions

In a world with growing pressures toward decarbonization, there is no longer a single, obvious business-as-usual” or “no policy reference” scenario. Instead, there is a range of plausible futures that should be explored. Researchers should think carefully about using a “business-as-usual” scenario and what it means. In particular, scenarios that continue historical trends of unfettered fossil fuel use no longer seem relevant when a shift toward a low-carbon society is already under way. We offer a scenario of a transition that reflects recent progress and pressures and how those might evolve at a natural pace into the future, absent globally coordinated mitigation effort. We consider a world that continues to address climate change in the way it has so far—through piecemeal actions and growing social awareness and technological advances.

This scenario takes into account the increasingly visible impacts of climate change that result in growing activism and demand for transition away from fossil fuels. Increasing pressure from voters, shareholders, consumers and investors prompt action by governments and businesses, steering investments away from fossil fuels. Technology and infrastructure investments and developments in renewables, energy storage, electrification, hydrogen and digitalization further push the low-carbon transition. In the scenario, these persistent and mounting pressures drive a series of plausible actions that lead to a transition that brings the global energy system to near zero emissions, and results in a global temperature increase of about 3 °C above preindustrial levels.

The plausible actions involved in our scenario bring society closer to the long-term goals of the Paris Agreement. They also provide a roadmap of ways in which the transition could be accelerated to bring the Paris goals within reach, and provide insight into the additional actions needed. Refined oil is highlighted as playing a particularly impactful role in the transition. Action in the forms of R&D, technology deployment, infrastructure development, policy incentives and business practices will all be essential to speed up the transition away from refined oil. Similarly, actions to help accelerate the decarbonization of energy intensive industries are needed.

A slower energy transition than imagined in our analysis for developing countries (such as African countries or India) is a real possibility. However, enabling an even faster transition in such countries is necessary for the Paris targets to be attainable. Global coordination to help such countries achieve important development goals in a sustainable way will therefore be critical. It is not enough to lead by example, it is necessary to transfer knowledge and available technologies to developing countries. Stepping up government actions, including deploying carbon pricing, supporting natural and geological carbon sinks, and facilitating lifestyle changes, are crucial for all regions of the world in order to accelerate the transition.

## Supplementary Information

Below is the link to the electronic supplementary material.

Supplementary file1 (DOCX 1553 kb)

## Footnotes

1 While the term “phase out” can be interpreted as a deliberate and forced reduction, here we use it in the modelling sense in that we have phased out certain energy types in the model as supported by our narrative in Sect. 2, which makes assumptions as to what drives the phase outs (e.g. falling costs of competing technologies, government regulations, industry/business commitments, consumer pressure, financing challenges, etc.).

2 Although the *GP_FastOil* scenario has a lower temperature outcome than *Growing Pressures*, that scenario is considered as requiring action beyond what we consider in the *Growing Pressures* narrative. It is therefore a scenario between *Growing Pressures* and 2C.

## References

[CR1] BP (2019) BP energy outlook—2019 Edition

[CR2] Chen Y-HH, Paltsev S, Reilly J, Morris J, Babiker M (2016). Long-term economic modeling for climate change assessment. Econ Model.

[CR3] Fajardy M, Morris J, Gurgel A, Herzog H, MacDowell N, Paltsev S (2021) The economics of bioenergy with carbon capture and storage (BECCS) deployment in a 1.5 °C or 2 °C world. Global Environ Change 68:102262

[CR4] Grant N, Hawkes A, Napp T, Gambhir A (2020). The appropriate use of reference scenarios in mitigation analysis. Nat Clim Chang.

[CR5] Hausfather Z, Peters G (2020). Emissions—the ‘business as usual’ story is misleading. Nature.

[CR6] IEA (2019). World energy outlook.

[CR7] IMF (2019) World economic outlook. International Monetary Fund

[CR8] IPCC (2014) Climate change 2014: synthesis report. In: Contribution of working groups I, II and III to the Fifth Assessment Report of the Intergovernmental Panel on Climate Change [Core Writing Team, R.K. Pachauri and L.A. Meyer (eds.)]. IPCC, Geneva, Switzerland

[CR9] IPCC (2018) Global warming of 1.5 °C

[CR10] Kapsalyamova Z, Paltsev S (2020). Use of natural gas and oil as a source of feedstocks. Energy Econ.

[CR11] Libardoni A, Forest C, Sokolov A, Monier E (2018). Advances in statistical climatology. Meteorol Oceanogr.

[CR12] MIT Joint Program (2021) Global change outlook. Massachusetts Institute of Technology. https://globalchange.mit.edu/publications/signature/2021-global-change-outlook

[CR13] Morris J, Farrell J, Kheshgi H, Thomann H, Chen H, Paltsev S, Herzog H (2019). Representing the costs of low-carbon power generation in multi-region multi-sector energy-economic models. Int J Greenhouse Gas Control.

[CR14] Morris J, Reilly J, Chen Y-HH (2019). Advanced technologies in energy-economy models for climate change assessment. Energy Econ.

[CR15] Morris J, Kheshgi H, Paltsev S, Herzog H (2021). Scenarios for the deployment of carbon capture and storage in the power sector in a portfolio of mitigation options. Clim Change Econ.

[CR16] Morris J, Reilly J, Paltsev S, Sokolov A (2021b) Representing socio-economic uncertainty in human system models. MIT Joint Program Report 347. http://globalchange.mit.edu/publication/17576

[CR17] Narayanan BG, Aguiar A, McDougall R (2012) Global trade, assistance, and production. The GTAP 8 Data Base

[CR18] Paltsev S, Reilly JM, Jacoby HD, Eckaus RS, McFarland J, Sarofim M, Asadoorian M, Babiker M (2005) The MIT emissions prediction and policy analysis (EPPA) model: version 4. MIT Joint Program Report 125 http://globalchange.mit.edu/publication/14578

[CR19] Paltsev S, Morris J, Kheshgi H, Herzog H (2021). Hard-to-abate sectors: the role of industrial carbon capture and storage (CCS) in emission mitigation. Appl Energy.

[CR20] Paltsev S, Gurgel A, Morris J, Chen H, Dey S, Marwah S (2021b) Economic analysis of the hard-to-abate sectors in India. In: MIT joint program on the science and policy of global change, Report 355. http://globalchange.mit.edu/publication/17673

[CR21] Shell (2018) Shell scenarios: sky—meeting the goals of the Paris agreement

[CR22] Shell (2021) The energy transformation scenarios

[CR23] Sokolov A, Kicklighter D, Schlosser CA, Wang C, Monier E, Brown-Steiner B, Prinn R, Forest C, Gao X, Libardoni A, Eastham S (2018). Description and evaluation of the MIT earth system model (MESM). AGU J Adv Model Earth Syst.

[CR24] UN (2015) Paris Agreement: http://unfccc.int/files/essential_background/convention/application/pdf/english_paris_agreement.pdf

[CR25] UN (2019) World population prospects. Department of Economic and Social Affairs. Population Division

