# Supplementary material for: Future energy: in search of a scenario reflecting current and future pressures and trends
Source: Environ Econ Policy Stud. 2022 Feb 16;25(1):31–61. doi: 10.1007/s10018-021-00339-1 (PMC8853390; doi:10.1007/s10018-021-00339-1)
Supplement: Supplementary file 1 — Supplementary file1 (DOCX 1553 kb) [file 10018_2021_339_MOESM1_ESM.docx]

**Supplementary Material**

## **S.1. Storyline Behind the Growing Pressures Scenario**

The trend throughout the 2010s of falling costs for renewable energy deployment continues through to the middle of the century. Governments at federal, state and city level remain under constant pressure from activists, NGOs and climate concerned voters to back this technology pathway through mandates, some modest pricing mechanisms in some countries, grid infrastructure and direct incentives (e.g. tax credits) for deployment. A plateau is eventually reached in scale, technology, underlying science and unit efficiency. While the pace of change may not be at the rates seen in the 2010s, by 2050, the cost of renewable energy deployment should fall by at least a further 25% when compared with 2020. Large scale solar PV and offshore wind rollout in the range of two to three cents per kilowatt hour would be commonplace. A similar trend emerges with energy storage, driven by necessity given the renewables deployment and employing a diverse range of technologies, bringing the system cost for electricity generation to a level that competes with natural gas in many circumstances and locations.

During the 2050s and 2060s a spate of new energy storage technologies emerges, catalysed by a burgeoning aviation industry (that has long overcome the 2020 pandemic impacts) that is under intense societal pressure to manage emissions and is looking to utilise electricity beyond very short haul routes. Scale up in the 2070s in combination with solar PV modules brings the system cost of electricity generation down further, outcompeting all other forms of generation.

But cost is not the only driver of transformation in the electricity system. During the 2020s, just a handful of new coal fired power stations can gain both regulatory approval and financial backing in OECD countries, but with the arrival of the 2030s this ends. With rising renewables deployment, dispatch of thermal power stations becomes increasingly problematic going into the 2030s and 2040s, undermining the economics of these units. By 2070 as age also takes its toll on existing facilities, the last remaining coal fired power stations in the OECD are closed.

Coal remains a popular investment in China, India, Africa and parts of Asia right through the first half of the century, but financing becomes increasingly problematic and renewable alternatives compete. The clean air experienced in many cities during the COVID-19 crisis creates pressure on new developments in the early 2020s, which continues to build as environmental and climate impacts become more discernible. The late 2040s see the last of the new coal fired power stations built in these regions and age inevitably starts to take its toll on the installed fleet. By the early part of the 2090s coal has almost completely vanished from the global electricity mix and by 2100 it has departed the scene.

One technology to manage emissions that doesn’t emerge at scale is carbon capture and storage (CCS). Although the technology matures and some projects may be developed over the years, the lack of a focused policy approach combined with the increasing competitiveness of renewable energy means that extensive use of CCS never eventuates. But it remains a viable alternative for niche industrial applications where demand for low carbon footprint products is important.

Natural gas progresses through a similar transition, but the driver for change is more cost based with system costs for renewables and storage falling sufficiently to compete. Natural gas continues to be utilised for grid stability but over time load factors fall to such an extent that new facilities are hardly built. Eventually the grid relies on older gas facilities running for a small number of hours per year. By 2050 there is little new natural gas investment in OECD countries and during the 2070s new natural gas investment diminishes globally, both for generation and new production fields. Natural gas use in industry extends into the 22^nd^ century but by 2130 is ultimately limited to petrochemical feedstock for its carbon molecules.

The increasing availability of competitive renewable power generation and energy storage, the price stability it offers, and the underlying corporate social responsibility push to reduce emissions through the purchase of renewable electricity leads to an increasing trend towards electrification throughout society. In some locations the trend towards electricity is also assisted by favourable policy developments such as renewable energy tax benefits, pushed by climate activists and voters.

As the climate warms and many more cities experience extreme temperature days (>40°C) in the summer, with a worrying but growing handful also experiencing very extreme temperatures (45-50°C) at least once a summer, air conditioning becomes very popular. Dual cooling and warming electric systems dominate for reasons of cost and convenience, which progressively removes natural gas and heating oil from domestic use. By the 2060s home heating is electric almost everywhere, with biomass use in some locations (e.g. rural Sweden). Convenience also plays a role, with electric induction stovetops for cooking and efficient heat pumps for managing temperature in homes both entering widespread use. Combustion of fuels in homes begins to disappear in many urban settings from the 2040s onwards.

From 1960 to 2020 the global rate of electrification was steady at about 2% points per decade, leading to 20% of electricity in final energy by 2020. During the 2020s this starts to increase and passes 3% points per decade by 2040. By the 2070s the increase has reached 4% points per decade. It means that by 2150, electricity makes up over two thirds of the global final energy mix.

An important mid to long term driver for industrial electrification comes from industrial companies in the EU facing both national legislation and regional directives to reach net-zero emissions by 2050 as well as needing to achieve their own corporate pledges. Although varying levels of commitment by successive governments lead to waxing and waning of the pressure over time, widespread efforts to electrify various processes or convert them to hydrogen-based systems lead to processes such as hydrogen smelting of iron ore. Large-scale hydrogen production via electrolysis using renewable energy becomes a compelling choice for meeting net-zero targets in the 2030s and 2040s, accelerating the trend, building on a significant German stimulus emerging from the pandemic recovery. As renewable electricity becomes cheaper and extensively deployed globally, these innovations are adopted more widely, leading to substantial industrial change in OECD countries through the 2070s and eventually throughout the world by 2110 as existing production facilities are revamped and renewed and new ones built under a more carbon aware financial framework. Increased consumer demand for green products also drives this process, a trend that started prior to 2020 with companies such as Apple using only recycled aluminium.

A further important catalyst was the decision in the early 2020s by the London Metal Exchange to launch a platform to trade ‘low-carbon’ aluminium mostly produced with renewable energy, marking the first time a metal is traded based on its environmental footprint in the exchange’s 143-year history (Financial Times, 2020). This trend is expanded to other metals and commodities over the ensuing years and the early price premium that emerges encourages big producers in India, China and the Middle East to install suitable facilities.

One area where electricity comes into its own is surface transport. Throughout the 2020s passenger vehicle manufacturers introduce a wide variety of models and compete on vehicle range and size. As a result, the uptake of electric vehicles begins to take shape. This is helped by some countries introducing phase out incentives for older internal combustion vehicles if the switch to electric is made and others introducing bans on the sale of internal combustion engine cars at various points from 2035 to 2050. Cities help the process by introducing low emission then zero emission driving zones in key areas. The C40 group of cities becomes increasingly bold in its actions as the market delivers the solutions they need. In 2025 London restricts the Square Mile to electric vehicles only, but quickly expands the zone in the years that follow.

But it isn’t until the late 2040s when a tipping point is reached as manufacturers start deciding to exit the internal combustion market completely, reminiscent of the General Motors decision to abandon right-hand drive vehicle manufacturing in 2020. The lower manufacturing costs for all parts of the vehicle except the battery, simplicity of after sales service and much smaller work forces linked to the R&D process leads to these decisions. During the 2070s internal combustion engine vehicle sales end globally and the era of these vehicles’ drifts to a close.

Road freight transport is more difficult to dislodge. The weight and cost of a battery required for long distance haulage and the time required for recharging limits electricity to urban heavy transport, such as buses, delivery vans and municipal vehicles. Electricity continues its creep into the urban sector, pushed by city authorities extending low emission zones and by innovative companies offering an increasing range of electric options. The same is true for agriculture, with electric farm machinery becoming pervasive in OECD countries by 2050 (John Deere, 2020). Although there are few internal-combustion based urban trucks, vans and farm vehicles remaining by the 2070s, the long-distance haulage fleet has remained largely in the fuel world, but particularly in regions where long-distance haulage is commonplace. Nevertheless, there is some electric creep into this sector as well as battery technology improves.

In the 2030s and 2040s as electric cars begin to deploy at scale into the passenger vehicle market, biofuels that are released from that market due to reduced E10-gasoline (i.e., gasoline with 10% blending of ethanol) demand move into the diesel fuel market, albeit with additional conversion processing. This helps to reduce emissions in the haulage sector and provides an important boost for the biofuel industry. But public pressure leads to increasing scrutiny of biofuel crops, with NGOs making use of AI and satellite data to effectively monitor global crop coverage, which in turn limits the overall biofuel fuel pool. So, this pathway undergoes change as well.

Haulage companies respond to both city emission restrictions and companies shifting their supply chains to a zero-emission base for reasons linked to corporate social responsibility and consumer demand. Electrification creeps into the lighter end of the road haulage fleet and through the 2030s and 2040s hydrogen fuel cell trucks begin to appear for larger vehicles, with early fuel networks appearing in California and Germany. But the transition is slow, and it is not until much later in the century that electricity and hydrogen dominate the haulage sector.

After the COVID-19 crisis in 2020 the aviation industry begins its path to recovery and climate activism promptly resumes. Because of the pandemic, new investment is initially limited and the anticipated early transition to electric planes for short haul use does not emerge. But as the industry resumes its growth path and activism prevents major airport expansion, short haul flights with under 100 passengers come under pressure to move away from major hubs due to a lack of landing and take-off slots, leading to a boom in smaller regional airports linked to hub satellite airports. This is ideal territory for the emergence of a cost competitive and viable commuter electric plane in the 2030s, although its initial impact on jet fuel demand is minimal. Nevertheless, a few smaller airframe manufacturers appear, and a competitive market develops, progressively improving on passenger capacity, comfort and range. Later in the 2040s these smaller companies become targets for the likes of Boeing and Airbus.

But there is no shift in emissions on long-haul flights until much later in the century when a variety of technologies merge to deliver new thinking on air frame design and propulsion. This arrives at a timely moment as the cost of supplying high grade hydrocarbons from crude oil for one sector with a very specific requirement is starting to become burdensome. This marks the beginning of the end for crude oil exploration, extraction, refining and shipping. The first of a new generation of planes appear in 2080 and by 2130 the global aviation fleet is completely transformed.

By the early decades of the 22^nd^ century, even in a world where global climate action never accelerates to the pace required for the Paris Agreement, the last remaining energy use demand for fossil fuels is collapsing. The fossil fuel demand tail is a long one and stretches well into the century, but is hardly noticeable in terms of atmospheric impact.

## **S.2. MIT EPPA Model**

The Economic Projection and Policy Analysis (EPPA) model is a recursive-dynamic, multi-region, multi-sector general equilibrium model of the world economy (Chen et al., 2016; Paltsev et al., 2005). The version applied here includes an updated and expanded number of technology options. It was designed to develop projections of economic growth, energy transitions and anthropogenic emissions of greenhouse gases and air pollutants. The model projects economic variables (GDP, energy use, sectoral output, consumption, prices, etc.) and emissions of greenhouse gases (CO_2_, CH_4_, N_2_O, HFCs, PFCs and SF_6_) and other air pollutants (CO, VOC, NOx, SO_2_, NH_3_, black carbon, and organic carbon) from combustion of carbon-based fuels, industrial processes, waste handling, agricultural activities and land use change. Key assumptions driving these projections include labor productivity growth, population growth, technology costs, fossil fuel resource availability, elasticities of substitution, energy efficiency improvements and urban pollutant trends. We explore uncertainty in these assumptions in Morris et al. (2021b).

The MIT Integrated Global Systems Model (IGSM) (Sokolov et al., 2017) combines the EPPA model of human activity and the MIT Earth System Model (MESM) (Sokolov, et al., 2018) of the Earth’s physical and biological systems. It projects environmental conditions that result from human activity, including concentrations, temperature, precipitation, ice and snow extent, sea level, ocean acidity and temperature, and vegetation, among other outcomes. The IGSM allows assessment of the 21^st^ century temperature implication of different emissions pathways.

The EPPA model used in this paper is built on the Global Trade Analysis Project Version 8 (GTAP8) economic dataset (Narayanan et al., 2012) which provides a consistent representation of regional production, bilateral trade flows, and markets. Energy and land markets are supplemented with accounting in physical units. This economic data is augmented with additional information on advanced technologies, greenhouse gases and air pollutants emissions, taxes and details of selected economic sectors. The data are aggregated to 18 regions and multiple sectors, with additional detail in the energy sector added in the form of advanced technology alternatives that are incorporated using bottom-up engineering detail (see infographic on **Figure S1**). For this work, additional electricity-based technology options were added to the industrial and commercial transportation sectors, as well as for final and intermediate demand. The approaches for defining the costs and penetration of these advanced technologies are described in Morris et al. (2019a,b).

A challenge for complex models such as EPPA is evaluation of their performance. While individual parameters of the model can be estimated statistically or informed by econometric studies, the data needed to estimate the entire set of model parameters does not exist to apply conventional goodness-of-fit measures on sufficiently robust historical data. The EPPA model validation is discussed in Chen et al (2016), where the model was run from an earlier starting point (year 2000) to assess the behavior of the model in the historical period and to compare projections to actual data to evaluate performance.

**MIT Economic Projection and Policy Analysis (EPPA) Model**

**Multi-sector, multi-region computable general equilibrium (CGE) model of the world economy for energy, economy and emissions projections**

**
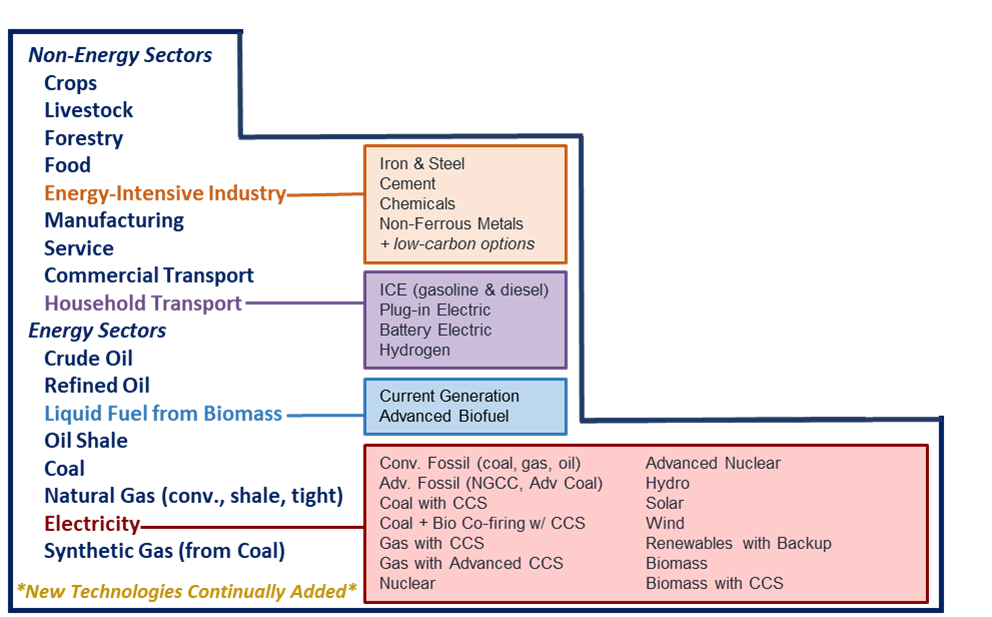
**

**Technical Features**

Written in GAMS^1^ using MSPGE^2^

Based on GTAP Data^3^

Calibrated to current economic and energy levels based on IMF^4^ and IEA^5^

Land-Use Component (crops. pasture, natural grass, natural forest, managed forest)

Documented in peer-reviewed literature^6-9^

Publicly Available Version^9^

2100+ (in 5-year steps)

[Grab your reader’s attention with a great quote from the document or use this space to emphasize a key point. To place this text box anywhere on the page, just drag it.]

Full Input-Output data

for every region

**
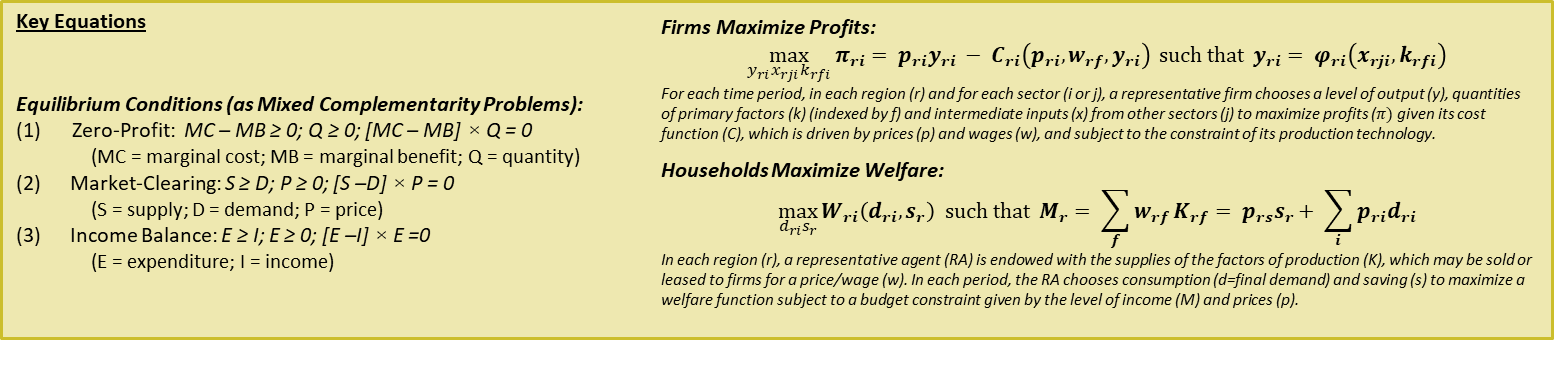
**

^1^ https://www.gams.com/; ^2^ https://www.gams.com/solvers/mpsge/index.htm; ^3^ https://www.gtap.agecon.purdue.edu/; ^4^ International Monetary Fund: https://www.imf.org/en/publications/weo; ^5^ International Energy Agency: https://www.iea.org/topics/world-energy-outlook; ^6^ Chen et al. (2016); ^7^ Morris et al. (2019a); ^8^ Morris et al. (2019b); ^9^ Paltsev et al. (2005); ^10^ https://globalchange.mit.edu/research/research-tools/human-system-model/download

**Figure S1.** Infographic of the EPPA Model.

The model’s production and consumption sectors are represented by nested Constant Elasticity of Substitution (CES) production functions (or the Cobb-Douglas and Leontief special cases of the CES) (see Chen et al., 2016; Paltsev et al., 2005 for deviations from this structure for specialized sectors such as oil refining and agriculture). The base year of the model is 2007. It is calibrated to economic and energy data from the IMF (2018) and IEA (2018) for 2010 and 2015 and then it is solved recursively in 5-year time steps from 2020 to 2100. The model is designed for projecting long-term trends, so it does not capture business cycles or short-term shocks such as those that often occur in, for example, commodity markets that play out over periods of less than the 5-year time step of the model.

A model solution must meet three conditions: market clearance conditions (supply must equal demand), zero profits (the cost of inputs should not exceed the price of the output), and income balance (expenditures must equal income, accounting for savings, subsidies and taxes). Production technologies are chosen based on their relative competitiveness given the characterization of input requirements for the technology, which, in turn, determine the cost of the technology given prices for the inputs. Base year prices for all inputs are in the base economic data of the model, and future technology costs depend on how prices change for inputs used by the technology. Input prices change over time depending on the dynamics of the model, including changes in the labor force, investment and capital availability, resource availability/depletion and existing or new policies or constraints. All technologies require capital and labor inputs, and so capital and labor productivity improvement (a major component of the dynamics of the model) drives down technology costs over time. The model also traces inter-industry and inter-regional demands. These influences combine to determine the resulting technology mix. As applied here, there are no environmental feedbacks on the economy.

An algebraic description of the major components of the model is below. The EPPA model is formulated as a mixed complementarity problem and uses the GAMS/MPSGE software to solve for equilibrium prices, quantities, and income levels. Our complementarity-based solution approach distinguishes price and demand equations, market clearance conditions, budget constraints, and auxiliary equations.

***Behavior of Firms***

In each region (indexed by the subscript r) and for each sector (indexed interchangeably by i or j), a representative firm chooses a level of output y, quantities of capital k and labor l, depletable and renewable resource factors (indexed by z) and intermediate inputs from other sectors j to maximize profits subject to the constraint of its production technology. Production is modeled assuming constant-elasticity-of-substitution (CES) functions that impose constant returns to scale. By duality and the property of linear homogeneity, optimizing behavior of the representative firm requires that:

$p_{r,i}=c_{r,i}\left(pa_{r,j},pl_{r},pk_{r},pr_{z}\right)$ (1)

where *p_r,i_, pa_r,j_, pl_r_, pk_r_, pr_z_* denote prices for domestic output, intermediate inputs, labor, capital, and resource factors, respectively. Function *c_r,i_* provides a generic representation of the unit cost function for sector *i*. A schematic overview of the adopted nesting CES structure for conventional production sectors and adopted nesting CES structure for production with advanced technologies is provided in Chen et al. (2016). Zero profits conditions in (1) exhibit complementary slackness with respect to the activity level *y_r,i_*. For each sector, ad-valorem sector- and region-specific output tax rates, denoted by *to_r,i_* enter at the top nest. Region-specific capital income tax rates, denoted by *tk_r_*, and payroll tax rates, denoted by *tl_r_*, enter in the value-added nest. Given input prices gross of taxes, firms maximize profits subject to technology constraints. Firms operate in perfectly competitive markets and sell their products at a price equal to marginal costs.

To illustrate how taxes enter the CES cost functions, consider the pricing equation for the agriculture sector. We write the equations in calibrated share form where *ɸ*’s denote respective benchmark value share parameters and an upper bar refers to the benchmark value of variables. The unit cost function is given by:

|  | $\frac{\left( \boldsymbol{1}-\boldsymbol{to}_{\boldsymbol{r},\boldsymbol{i}} \right)\boldsymbol{p}_{\boldsymbol{r},\boldsymbol{i}}}{\left( \boldsymbol{1}-{\bar{\boldsymbol{to}}}_{\boldsymbol{r},\boldsymbol{i}} \right){\overline{\boldsymbol{p}}}_{\boldsymbol{r},\boldsymbol{i}}}=\left[ \boldsymbol{\phi}_{\boldsymbol{r},\boldsymbol{RES},\boldsymbol{i}}\left( \frac{\boldsymbol{p}_{\boldsymbol{r},\boldsymbol{RES}}}{{\overline{\boldsymbol{p}}}_{\boldsymbol{r},\boldsymbol{RES}}} \right)^{\boldsymbol{1}-\boldsymbol{\sigma}_{\boldsymbol{EVRA}}}+\boldsymbol{\phi}_{\boldsymbol{r},\boldsymbol{VA},\boldsymbol{i}}\left( \frac{\boldsymbol{p}_{\boldsymbol{r},\boldsymbol{VA}}}{{\overline{\boldsymbol{p}}}_{\boldsymbol{r},\boldsymbol{VA}}} \right)^{\boldsymbol{1}-\boldsymbol{\sigma}_{\boldsymbol{EVRA}}} \right]^{\frac{\boldsymbol{1}}{\boldsymbol{1}-\boldsymbol{\sigma}_{\boldsymbol{EVRA}}}}$ | (2) |
| --- | --- | --- |

where *p_r,RES_* denotes the price for the resource-intensive input bundle and the price for the value-added composite, *p_r,VA_*, is given by:

$\frac{p_{r,VA}}{\overline{p}_{r,VA}}=\left[\varphi_{r,i,L}\left(\frac{\left(1+tl_{r}\right)pl_{r}}{\left(1+\overline{tl}_{r}\right)\overline{pl}_{r}}\right)^{1-\sigma_{VA}}+\varphi_{r,i,K}\left(\frac{\left(1+tk_{r}\right)pk_{r}}{\left(1+\overline{tk}_{r}\right)\overline{pk}_{r}}\right)^{1-\sigma_{VA}}\right]^{\frac{1}{1-\sigma_{VA}}}$ (3)

Elasticities are denoted by σ. The elasticity values are reported in Chen et al. (2016).

By Shephard's Lemma, the demand for good *j* by sector *i* is:

|  | $\boldsymbol{x}_{\boldsymbol{r},\boldsymbol{j},\boldsymbol{i}}=\boldsymbol{y}_{\boldsymbol{r},\boldsymbol{i}}\frac{\boldsymbol{\partial}\boldsymbol{c}_{\boldsymbol{r},\boldsymbol{i}}}{\boldsymbol{\partial}\boldsymbol{pa}_{\boldsymbol{r},\boldsymbol{j}}}$ | (4) |
| --- | --- | --- |

and the demand for labor, capital, and resource factors is:

|  | $\boldsymbol{ld}_{\boldsymbol{r},\boldsymbol{i}}=\boldsymbol{y}_{\boldsymbol{r},\boldsymbol{i}}\frac{\boldsymbol{\partial}\boldsymbol{c}_{\boldsymbol{r},\boldsymbol{i}}}{\boldsymbol{\partial}\boldsymbol{pl}_{\boldsymbol{r}}}$ | (5) |
| --- | --- | --- |
|  | $kd_{r,i}=y_{r,i}\frac{\partial c_{r,i}}{\partial pk_{r}}$ | (6) |
|  | $\boldsymbol{rd}_{\boldsymbol{r},\boldsymbol{z},\boldsymbol{i}}=\boldsymbol{y}_{\boldsymbol{r},\boldsymbol{i}}\frac{\boldsymbol{\partial}\boldsymbol{c}_{\boldsymbol{r},\boldsymbol{i}}}{\boldsymbol{\partial}\boldsymbol{pr}_{\boldsymbol{r},\boldsymbol{z}}}$ | (7) |

##

## ***Domestic and Foreign Trade***

We adopt the Armington assumption of product heterogeneity for imports and exports. Sectoral output produced in each region is converted through a CET function into goods destined for the national and international markets. The associated unit cost function is given by:

$\frac{p_{r,i}}{\overline{p_{r,i}}}=\left[\alpha_{r,i,d}\left(\frac{pd_{r,i}}{\overline{pd_{r,i}}}\right)^{1+\eta}+\alpha_{r,i,f}\left(\frac{pdfx_{r,i}}{\overline{pdfx_{r,i}}}\right)^{1+\eta}\right]^{\frac{1}{1+\eta}}$ **(**8)

where *pd_r,i_*, and *pdfx_r,i_* denote the price for domestic output, and international exports, respectively, and α’s are value shares parameters. All goods are tradable. Analogously to the export side, we adopt the Armington assumption of product heterogeneity for imports. A nested CES function characterizes the trade-off between imported (from international sources) and locally produced varieties of the same goods. The zero-profit conditions that determine the level of Armington production, denoted by *a_r,j_*, is given by:

$\frac{pa_{r,i}}{\overline{pa_{r,i}}}=\left[\beta_{r,d,i}\left(\frac{pd_{r,i}}{\overline{pd_{r,i}}}\right)^{1-\sigma_{DM}}+\beta_{r,f,i}\left(\frac{pdfm_{r,i}}{\overline{pdfm_{r,i}}}\right)^{1-\sigma_{DM}}\right]^{\frac{1}{1-\sigma_{DM}}}$ (9)

where *pdfm_r,j_* and *β*’s denotes the price for imports and respective value share parameters, respectively.

##

## ***Household Behavior***

In each region *r*, a representative household is endowed with the supplies of the factors of production, the services of which may be sold or leased to firms. In each period, the representative agent chooses consumption and saving to maximize a welfare function subject to the budget constraint given by the level of income *M*, which is defined as:

$M_{r}=\left(pk_{r}\cdot\overline{K}_{r}+pl_{r}\cdot\overline{L}_{r}\right)\left(1-tinc_{r}\right)+pk\cdot\overline{RK}_{r}+\sum_{z} pr_{z}\cdot\overline{F}_{r}+TR_{r}$ (10)

where $\overline{K}_{r}$, $\overline{L}_{r}$, $\overline{F}_{r}$, $\overline{RK}_{r}$denote the initial endowment of non-residential capital, labor, fossil fuel resources, and residential capital, respectively. $tinc_{r}$ and $\mathrm{tinc}_{r,h}$denote the region-specific marginal personal income tax rate and transfer income, respectively.

Preferences are represented by a CES function where consumption, labor supply, and savings resulted from the decisions of representative households in each region maximizing utility subject to a budget constraint. By duality and the property of linear homogeneity, optimizing behavior of households requires that:

$pw_{r}=E_{r}\left(pa_{r,i},pl_{r},pk_{r},pinv_{r}\right)$ (11)

where *pw_r_* denotes a utility price index. *pinv_r_* denotes the price for the investment good in region *r* which is produced with fixed production coefficients according to:

|  | $\frac{\boldsymbol{pinv}_{\boldsymbol{r}}}{{\overline{\boldsymbol{pinv}}}_{\boldsymbol{r}}}=\sum_{\boldsymbol{i}} \boldsymbol{\phi}_{\boldsymbol{r},\boldsymbol{i},\boldsymbol{INV}}\frac{\boldsymbol{pa}_{\boldsymbol{r},\boldsymbol{i}}}{{\overline{\boldsymbol{pa}}}_{\boldsymbol{r},\boldsymbol{i}}}$ | (12) |
| --- | --- | --- |

By Shephard's Lemma, the compensated final demand for good *i* by household in region *r* is given by:

$d_{r,i}=\overline{M}_{r}\frac{\partial E_{r}}{\partial pa_{r,i}}$ (13)

|  |  | |  |
| --- | --- | --- | --- |
|  | and investment demand is given by: $nrd_{r}=\overline{M}_{r}\frac{\partial E_{r}}{\partial pinv_{r}}$ | (14) | |

***Government***

The government is modeled as a passive entity, which collects taxes and distributes the receipts to the households. Provision of public goods is a CES aggregate of Armington goods whose price is given by:

|  | $\frac{\boldsymbol{pgov}_{\boldsymbol{r}}}{{\overline{\boldsymbol{pgov}}}_{\boldsymbol{r}}}=\left[ \sum_{\boldsymbol{i}} \boldsymbol{\xi}_{\boldsymbol{r},\boldsymbol{i}}\left( \frac{\boldsymbol{pa}_{\boldsymbol{r},\boldsymbol{i}}}{{\overline{\boldsymbol{pa}}}_{\boldsymbol{r},\boldsymbol{i}}} \right)^{\boldsymbol{1}-\boldsymbol{\sigma}_{\boldsymbol{GOV}}} \right]^{\frac{\boldsymbol{1}}{\boldsymbol{1}-\boldsymbol{\sigma}_{\boldsymbol{GOV}}}}$ | (15) |
| --- | --- | --- |

and where $\xi_{r,i}$ denote value shares parameters. The government budget constraint is given by:

|  | ${GOV}_{\boldsymbol{r}}=\sum_{\boldsymbol{i}} \left( \boldsymbol{to}_{\boldsymbol{i,r}}{\cdot\boldsymbol{p}}_{\boldsymbol{i,r}}{\cdot\boldsymbol{y}}_{\boldsymbol{i,r}}+\boldsymbol{tl}_{\boldsymbol{i,r}\cdot}\boldsymbol{pl}_{\boldsymbol{r}}{\cdot\boldsymbol{ld}}_{\boldsymbol{i,r}}+\boldsymbol{tk}_{\boldsymbol{i}}\cdot\boldsymbol{pk}_{\boldsymbol{r}}\cdot\boldsymbol{kd}_{\boldsymbol{i,r}} \right)+\boldsymbol{tinc}_{\boldsymbol{r}}\boldsymbol{(}\boldsymbol{pl}_{\boldsymbol{r}} \cdot{\overline{\boldsymbol{L}_{\boldsymbol{r}}}}\boldsymbol{+}\boldsymbol{pk}_{\boldsymbol{r}} ({{\overline{\boldsymbol{K}}}}_{r}\boldsymbol{-}\boldsymbol{nrd}_{\boldsymbol{r}}\boldsymbol{)})-\overline{\boldsymbol{BOP}_{\boldsymbol{r}}}$ | (16) |
| --- | --- | --- |

where $\overline{BOP}_{r}$ denotes the initial balance of payments (deficit).

## ***Market Clearing Conditions***

The system is closed with a set of market clearance equations that determine the equilibrium prices in the different goods and factor markets. The market clearance condition for Armington goods requires that:

|  | $\boldsymbol{a}_{\boldsymbol{r},\boldsymbol{i}}=\sum_{\boldsymbol{j}} \boldsymbol{x}_{\boldsymbol{r},\boldsymbol{i},\boldsymbol{j}}+\boldsymbol{d}_{\boldsymbol{r},\boldsymbol{i}}+\boldsymbol{i}_{\boldsymbol{r}}\cdot\frac{\boldsymbol{\partial}\boldsymbol{pinv}_{\boldsymbol{r}}}{\boldsymbol{\partial}\boldsymbol{pa}_{\boldsymbol{r},\boldsymbol{i}}}+\boldsymbol{g}_{\boldsymbol{r}}\cdot\frac{\boldsymbol{\partial}\boldsymbol{pgov}_{\boldsymbol{r}}}{\boldsymbol{\partial}\boldsymbol{pa}_{\boldsymbol{r},\boldsymbol{i}}}$ | (17) |
| --- | --- | --- |

By Shephard's Lemma, the two last summands in (17) represent the investment and government demand for good *i* in region *r*, respectively. Regional labor markets are in equilibrium if:

|  | ${\overline{\boldsymbol{L}}}_{\boldsymbol{r}} =\sum_{\boldsymbol{i}} \boldsymbol{ld}_{\boldsymbol{r},\boldsymbol{i}}$ | (18) |
| --- | --- | --- |

the capital market in region *r* clears if:

|  | $\left( {\overline{\boldsymbol{RK}}}_{\boldsymbol{r}}+\boldsymbol{K}_{\boldsymbol{r}} \right)=\sum_{\boldsymbol{i}} \boldsymbol{kd}_{\boldsymbol{r},\boldsymbol{i}}+\boldsymbol{rsd}_{\boldsymbol{r}}$ | (19) |
| --- | --- | --- |

and equilibrium on resource markets requires that:

|  | $\sum_{\boldsymbol{r}} {\overline{\boldsymbol{F}}}_{\boldsymbol{r},\boldsymbol{z}}=\sum_{\boldsymbol{i}} \boldsymbol{rd}_{\boldsymbol{r},\boldsymbol{z},\boldsymbol{i}}$ | (20) |
| --- | --- | --- |

Foreign closure of the model is warranted through a national balance-of-payments (BOP) constraint that determines the real exchange rate:

|  | $\sum_{\boldsymbol{i}} \boldsymbol{EX}_{\boldsymbol{r},\boldsymbol{i}}+{\overline{\boldsymbol{BOP}}}_{\boldsymbol{r}}=\sum_{\boldsymbol{i}} \boldsymbol{IM}_{\boldsymbol{i,r}}\frac{\boldsymbol{\partial}\boldsymbol{pdfm}_{\boldsymbol{i,r}}}{\boldsymbol{\partial pfx}_{\boldsymbol{r}}}$ | (21) |
| --- | --- | --- |

where *EX_r,i_*${\overline{\mathrm{EX}}}_{r,i}$, are exports and *IM_i,r_* are imports. The evolution of the economy and its energy-using characteristics over time are determined by the rate of capital accumulation, population and labor force growth, changes in the productivity of labor and energy, fossil fuel resource depletion, the availability of initially unused “backstop” energy supply technologies. Additional information about the dynamic structure of EPPA is provided in Chen et al. (2016).

## **S.3. Additional Figures and Tables**

**Figure S2.** Assumed population projections by region in the EPPA model.

**Figure S3.** Assumed population growth rates by region in the EPPA model.

**Figure S4.** Assumed GDP projections by region in the EPPA model.

**Figure S5.** Assumed GDP growth rates by region in the EPPA model.

**Figure S6.** CH_4_ and N_2_O trajectories for *Historic Trends*, *Growing Pressures* and *Shell SKY* scenarios. The trajectories shown for *Growing Pressures* are used for *Scenario Element 9* (as described in the main text in Table 1).

**Table S1.** Assumed actions driving emissions reductions, including for methane (CH_4_) and Nitrous Oxide (N_2_O), in the Shell *SKY* scenario. These actions drive the Shell *SKY* CH_4_ and N_2_O trajectories shown in Figure S6.

| Gas | Sector | Actions assumed between 2020-2070 |
| --- | --- | --- |
| Carbon dioxide | Cement | - Progressive substitution away from cement in buildings - Some substitution away from limestone as a feedstock, e.g., using fly-ash - Using carbon capture and storage (CCS) |
|  | Industrial (process emissions) | - Using CCS |
|  | Agriculture | - Eliminating deforestation for land gain - Implementing soil carbon programmes, e.g., no-till farming, land-use rotation |
|  | Urbanisation and development | - Creating green cities through extensive tree planting - Maintaining green belts within and around cities - Avoiding city spread through higher density living - Addressing traditional biomass usage through modern access to energy programmes |
| Methane | Coal mining | - Reducing coal consumption - Implementing best practice for methane drainage and use in coal mines (e.g., UNECE Guidance) - Managing abandoned mines |
|  | Oil and gas industry | - Reducing oil and gas consumption - Oil and gas industry leaders implementing best-practice from the 2020s, and all world production meeting best-practice by 2050 |
|  | Cattle farming | - Offering alternative products to consumers - Changing cattle diets to minimise methane |
|  | Rice growing | - Reducing forced flooding in rice paddies |
|  | Urbanisation and development | - Capturing methane from landfill |
| Nitrous oxide | Agriculture | - Implementing nitrogen fertiliser management, i.e., application rate, formulation (fertiliser type), timing of application, placement |
|  | Industrial processes | - Implementing catalytic decomposition and thermal destruction techniques |
| Fluorinated gases | Various  (e.g., IT industry, refrigeration, transformers) | - Progressive substitution away from PFC, HFC, and SF_6_ - Using best practice management - Introducing recovery programmes for retired equipment (e.g., refrigerators, transformers) |
